# Aberrant hepatic glycogenolysis and elevated insulin clearance can lead to major glucose irregularities in type 2 diabetes: insights from system identification

**DOI:** 10.3389/fphys.2026.1907805

**Published:** 2026-08-26

**Authors:** Chengcheng Wang, Jill A. Kanaley, Junqing Chen, Jinglu Tan

**Affiliations:** Division of Food, Nutrition & Exercise Sciences, University of Missouri, Columbia, MO, United States

**Keywords:** diabetes, endogenous glucose production, exercise timing, glucose regulation, obesity, system identification

## Abstract

Complex interactions obscure the behavior of the glucose regulation processes as a system, hampering advances in diabetes care. We synthesized the current understandings of pancreatic hormones, glucose transporters, and glycogen metabolism into a *model structure* of biochemical reactions and mass transport. The model structure was then used as a *soft sensor* or filter to computationally extract system characteristics (model coefficients) from experimental measurements. Changes in coefficient values were analyzed to observe system differences among non-obese (n=18), obese (n=18), and type 2 diabetic (n=16) individuals under three conditions: none, morning, and post-dinner exercise. The model structure captures the major kinetics in blood glucose, insulin, and glucagon and the extracted coefficient values revealed metabolic differences and impairments. Relative to the non-obese group, the obese group showed impaired glucagon clearance (47% reduction) and reduced insulin sensitivity for glycogenesis, while the diabetic group exhibited elevated glucagon sensitivity (2.4-fold increase) and an aberrant effect of glycogen on glycogenolysis, which was associated with dysregulated hepatic glucose production and rising glucose during fasting. Parameter sensitivity analysis identified insulin clearance as a dominant factor in endogenous glucose production and fasting glucose level. Exercise effects varied: post-dinner exercise enhanced hepatic glycogen synthesis and hepatic sensitivity to glucose in non-obese individuals, while morning exercise improved muscle glucose uptake in the groups with obesity and type 2 diabetes. Derivation of the model structure and its application as a soft sensor elucidate the complex metabolic interactions underlying diabetes and provide a method to generate hypothesis for future research.

## Introduction

Blood glucose homeostasis is crucial for maintaining cellular function and energy balance in mammals, ensuring that blood glucose levels remain within a narrow physiological range of approximately 70–110 mg/dL in humans despite fluctuations in nutritional states and energy demands (Saltiel and Kahn, 2001; Röder et al., 2016). Disruptions to this tightly controlled system can lead to metabolic disorders, such as diabetes mellitus, characterized by chronic hyperglycemia and its associated complications.

The regulation of blood glucose involves a complex cascade of biochemical signals, including hormones, enzymes, and transporter proteins, which act in concert across multiple tissues. Insulin and glucagon, two key pancreatic hormones, play opposing roles as primary regulators: insulin facilitates glucose uptake and utilization in peripheral tissues in the fed state, while glucagon promotes hepatic glucose production during fasting (Marin-Penalver et al., 2016; Röder et al., 2016).

At the tissue level, the liver, skeletal muscles, and adipose tissues serve as the primary sites for glucose uptake, utilization, and storage. These tissues have distinct glucose transport mechanisms and metabolism characteristics: hepatocytes predominantly express GLUT2 transporters, which facilitate bidirectional glucose movement and function independently of insulin, allowing the liver to play a central role in both glucose uptake and release (Thorens and Mueckler, 2010). In contrast, skeletal muscles and adipose tissues express GLUT4 transporters, which are translocated to the cell membrane in response to insulin signaling, promoting glucose uptake particularly in postprandial states (Thorens and Mueckler, 2010; Richter and Hargreaves, 2013). Glucose taken up by the liver and skeletal muscles is primarily stored as glycogen. When glycogen storage capacity is exceeded and energy intake surpasses expenditure, excess glucose is diverted to *de novo* lipogenesis, where it is converted into fatty acids and subsequently stored as triglycerides in adipose tissue (Postic and Girard, 2008).

During exercise, muscle glucose utilization increases significantly through both insulin-dependent and insulin-independent mechanisms, highlighting the adaptability of glucose metabolism to varying physiological demands (Jensen and Richter, 2012; Richter and Hargreaves, 2013). These intricate interactions make glucose kinetics very complex. Conventional statistical analysis of data can reveal important local trends and effects but are often ineffective in painting a holistic picture from multidimensional experimental data with nonlinear interactions. Mathematical modeling is an effective tool to synthesize existing knowledge and interpret experimental data for insights into the system behavior under different conditions.

Several mathematical models have previously addressed different aspects of glucose metabolism, ranging from minimal models that focus only on insulin-glucose dynamics (Bergman et al., 1979) to more comprehensive models incorporating multiple tissues and metabolic pathways (Dalla Man et al., 2006; Li et al., 2006; Maas et al., 2006; Man et al., 2007; Cobelli et al., 2014; Alkhateeb et al., 2021). However, most existing models are either based on much simplified tissue-specific mechanisms or focused on specific physiological states.

In this research, we synthesized and represented the existing knowledge on hormonal regulation, glucose transport, and glycogen metabolism in both liver and muscle tissues in the form of an extended kinetic model structure. The major reaction or transport rates were formulated based on the underlying biological processes. The physiologically constrained kinetic model structure was then used as a computational soft sensor or filter to estimate physiological coefficients from experimental data for individual subjects in varied health and exercise conditions. By combining mechanistic model structure with system identification, this work aims to shed light on the physiological process interactions underlying metabolic dysfunction and exercise responses.

## Materials and methods

### Subjects and protocol

The study was approved by the University of Missouri Health Sciences Institutional Review Board (Protocol #1202202) adhering to the Declaration of Helsinki, and was registered at ClinicalTrials.gov (NCT03019510; Jan 10, 2017). Written informed consent was obtained from all participants. Fifty-two adults aged 25–65 years were enrolled and classified into three groups: 18 non-obese (BMI<25 kg/m^2^), 18 obese (BMI 30–45 kg/m^2^), and 16 obese with impaired fasting glucose. All participants were weight-stable, non-smokers, and had no history of bariatric surgery. The Nonobese and Obese groups had normal fasting glucose levels (<100 mg/dL), while the obese group with impaired fasting glucose were previously diagnosed with type 2 diabetes (9 participants) or had fasting glucose >110 mg/dL on four of seven screening days and are labeled as the Diabetic group. Although the diabetic participants were not using insulin, all were on at least one glucose-lowering medication, with many also taking statins and antihypertensives. In contrast, the Obese and Nonobese participants were not on glucose-lowering drugs, and only a few used statins or antidepressants. Women taking oral contraceptives were assessed during the active pill phase, and pregnant individuals were excluded. Baseline participant characteristics are summarized in Table 1 in Kanaley et al. (2024).

The study followed a randomized crossover design in which all participants completed three conditions: no exercise (NOEX), morning exercise at 07:00 h (AMEX), and evening exercise at 20:00 h (PMEX). Exercise was supervised and VO_2_ was monitored to maintain target intensity. AMEX sessions occurred at 07:00 h on the study day, while PMEX sessions were at 20:00 h, two hours post-dinner. NOEX participants refrained from exercise for 48 hours before the study. The order of study conditions was counterbalanced, and at least three weeks separated each condition. More details of the experimental design are in (Kanaley et al., 2024).Blood sample collection began at 17:40 h and continued through 07:00 h the next morning. Samples were drawn into EDTA tubes (with and without DPP-IV inhibitor and aprotinin), chilled, centrifuged, aliquoted, and stored at −80 °C. Glucose was measured with a YSI 2300 STAT PLUS, and insulin and glucagon concentrations were determined by using the Milliplex Human Metabolic Hormone Panel (MilliporeSigma). Plasma samples collected at 00:00 h and 06:00 h were analyzed for [6,6-^2^H_2_] glucose enrichment by gas chromatography–mass spectrometry.

### Model structure formulation

**Figure 1:**
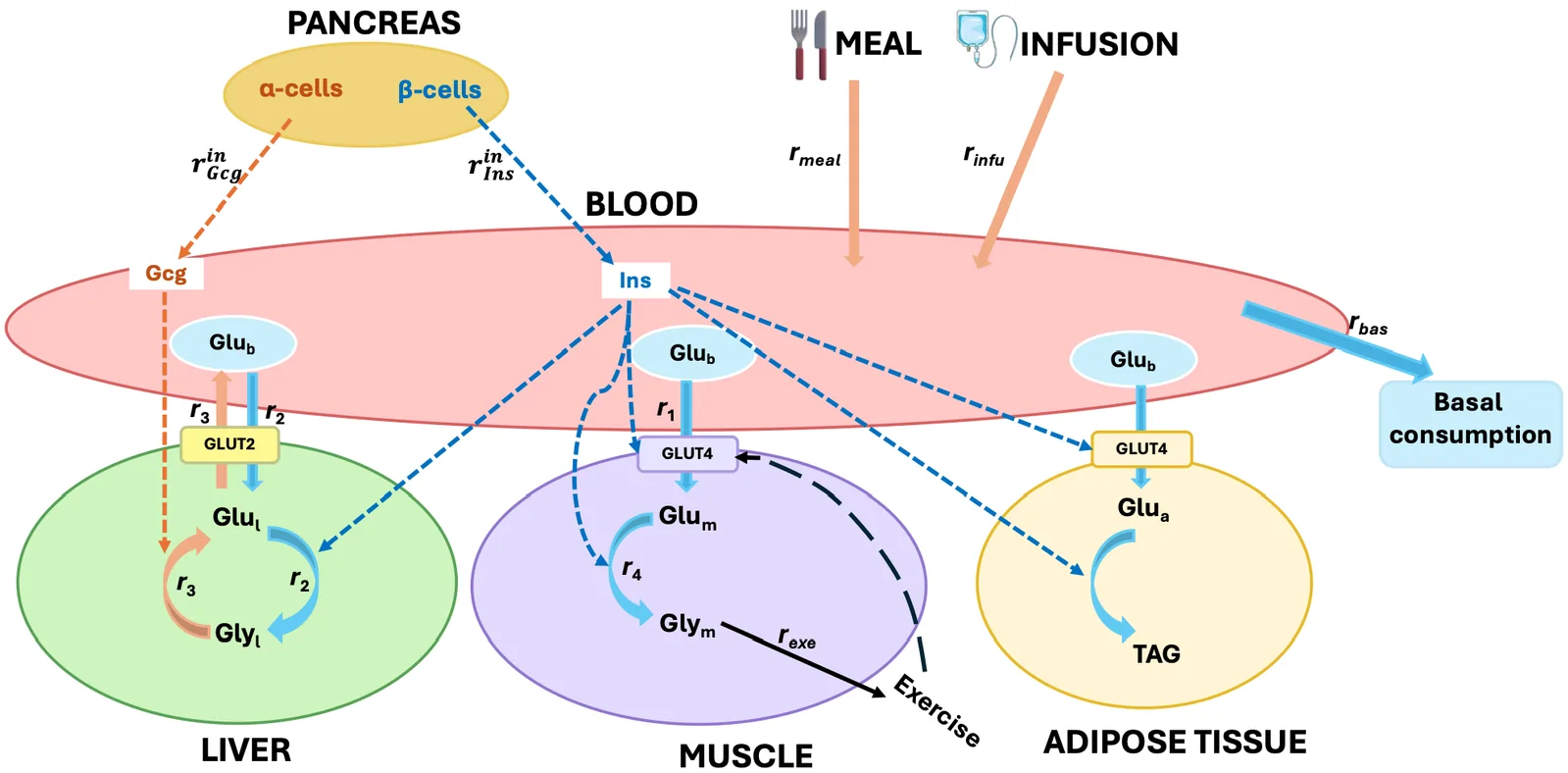
Major fluxes, rates, and signals in the glucose–insulin–glucagon regulatory system for blood glucose. Solid arrows represent metabolic fluxes between compartments, while dashed arrows denote hormonal signaling pathways. Glucose enters the bloodstream through dietary intake (*r_meal_*), intravenous infusion (*r_infu_*), and hepatic glycogenolysis (*r*_3_). Clearance of blood glucose occurs via muscle uptake (*r*_1_) mediated by GLUT4, liver uptake (*r*_2_) mediated by GLUT2, basal metabolic consumption (*r_bas_*), and exercise demand (*r_exe_*), which also modulates GLUT4. *De novo* lipogenesis is not included because it is much slower and only influences long-term system behaviors. The pancreas regulates circulating glucose levels by secreting insulin (*Ins*) from β-cells and glucagon (*Gcg*) from α-cells in response to blood glucose concentration (*Glu_b_*). The time rates of change (time derivatives *d*/*dt*) of insulin (*Ins*), glucagon (*Gcg*), blood glucose (*Glu_b_*), muscle glucose (*Glu_m_*), liver glycogen (*Gly_l_*), and muscle glycogen (*Gly_m_*) are expressed in input and output rates. Development of these equations is detailed in the text and parameter definitions are summarized in **Table 1**.

**Figure 1** depicts the major physiological components, rates, and fluxes involved in the regulation of glucose metabolism. To represent blood glucose concentration as the system variable of interest (output), blood can be taken as the object of observation (control volume) into or out of which glucose is transported via different fluxes (inputs and outputs) mediated by insulin, glucagon, and other signals. The kinetic status of the system may be represented by six major variables (system states):

▪ Blood insulin concentration (*Ins*),
▪ Blood glucagon concentration (*Gcg*),
▪ Blood glucose concentration (*Glu_b_*),
▪ Muscle glucose concentration (*Glu_m_*),
▪ Muscle glycogen concentration (*Gly_m_*), and
▪ Liver glycogen concentration (*Gly_l_*).

As inputs into the control volume (blood), glucose enters the bloodstream through dietary intake (*r_meal_*), intravenous infusion (*r_infu_*), and hepatic glycogenolysis (*r*_3_) which is primarily modulated by glucagon. Glucose exits from blood via muscle uptake (*r*_1_) facilitated by glucose transporter GLUT4 that is modulated by insulin, liver uptake (*r*_2_) facilitated by GLUT2, basal metabolic consumption (*r_bas_*), and exercise consumption (*r_exe_*), which also modulates GLUT4. *De novo* lipogenesis is not included here because it is much slower than the overnight kinetics measured in this work and only influences the system in long-term observations (Postic and Girard, 2008). The pancreas secrets insulin (*Ins*) from β-cells and glucagon (*Gcg*) from α-cells in response to blood glucose concentration (*Glu_b_*). These activities form a closed-loop system. The major rates, fluxes, and signaling pathways involved are described below.

### Hormonal signaling

#### Insulin

Insulin production and clearance are essential for maintaining glucose homeostasis and are tightly regulated by blood glucose levels. After nutrient intake, elevated blood glucose concentrations stimulate insulin secretion from pancreatic β-cells, thereby promoting glucose uptake and storage in peripheral tissues such as muscle and liver. This secretion process exhibits a nonlinear, sigmoidal dependence on blood glucose, often captured by using a Hill-type function (Topp et al., 2000). Insulin clearance can be represented as a first-order process proportional to its concentration in blood according to experimental observations (Polonsky and Rubenstein, 1984; Henquin, 2000; De Gaetano et al., 2008). The overall rate of increase of blood insulin is (secretion minus clearance)


$$r_{Ins}=r_{Ins}^{in}-r_{Ins}^{out}=\frac{k_{1}Glu_{b}^{k_{3}}}{k_{2}^{k_{3}}+Glu_{b}^{k_{3}}}-k_{4}Ins$$

where *k*_1_ represents the maximum insulin secretion rate, *k*_2_ is the glucose concentration at which secretion is half-maximal, *k*_3_ is the Hill coefficient, and *k*_4_ is the insulin clearance rate constant.

#### Glucagon

Glucagon is a key counter-regulatory hormone secreted by pancreatic α-cells, playing an essential role in maintaining glucose homeostasis, especially during fasting or hypoglycemia. Its secretion is inversely regulated by blood glucose levels: stimulated when glucose is low and suppressed when glucose is high. This inverse relationship has been demonstrated in both experimental and clinical studies and can be mathematically represented by using a Hill-type function that captures the nonlinear suppression of glucagon production by glucose (Rorsman et al., 1989; Gylfe and Gilon, 2014). Glucagon clearance follows first-order kinetics as has been validated in multiple studies (Morettini et al., 2021; Huard and Kirkham, 2022). The overall rate of change in blood glucagon concentration is


$$r_{Gcg}=r_{Gcg}^{in}-r_{Gcg}^{out}=\frac{k_{5}k_{6}^{k_{7}}}{k_{6}^{k_{7}}+Glu_{b}^{k_{7}}}-k_{8}Gcg$$

where *k*_5_ denotes the maximum rate of glucagon secretion, *k*_6_ represents the glucose concentration at which secretion is half-maximally suppressed, *k*_7_ is the Hill coefficient, and *k*_8_ is the glucagon clearance constant.

### Glucose transporters

#### GLUT4-mediated glucose transport in muscle

Glucose transport in skeletal muscle is primarily mediated by glucose transporter 4 (GLUT4), a facilitative transporter that enables glucose to move across the muscle cell membrane driven by concentration gradient. This transport process can be quantitatively described by the Michaelis-Menten equation, which characterizes glucose uptake based on the transporter’s affinity for glucose and its maximal transport capacity (Katz et al., 1986; James et al., 1988; Cline et al., 1999; Shepherd and Kahn, 1999; Man et al., 2007).

Under basal conditions, most GLUT4s reside in intracellular vesicles, with only a small fraction present at the cell surface (Bryant et al., 2002). Upon insulin stimulation, a signaling cascade involving insulin receptor substrates (IRS), phosphatidylinositol 3-kinase (PI3K), and Akt/protein kinase B is activated, resulting in the translocation of GLUT4 to the plasma membrane (Huang and Czech, 2007; Jaldin-Fincati et al., 2017; Klip et al., 2019). This insulin-stimulated GLUT4 trafficking mechanism and subsequent glucose uptake have been modeled with a modified Michaelis-Menten framework, in which insulin concentration modulates transporter activity, and the transmembrane glucose gradient serves as the effective substrate.

In addition to insulin, skeletal muscle glucose uptake is also enhanced by exercise through insulin-independent pathways. Muscle contraction activates AMP-activated protein kinase (AMPK), Ca^2+^/calmodulin-dependent protein kinase (CaMK), and other signaling molecules, promoting GLUT4 translocation to the plasma membrane independently of insulin (Booth and Winder, 2005; Richter and Hargreaves, 2013; Sylow et al., 2017).

The insulin- and exercise-stimulated glucose uptakes yield an overall rate of glucose transport from blood to muscle as


$$r_{1}=\frac{k_{9}Ins\left(\;Glu_{b}-Glu_{m}\right)}{k_{10}+\left(Glu_{b}-Glu_{m}\right)}+\frac{k_{11}\left(\;Glu_{b}-Glu_{m}\right)}{k_{12}+\left(Glu_{b}-Glu_{m}\right)}$$

where *k*_9_ *Ins* represents the maximum rate of muscle glucose uptake modulated by insulin concentration with *k*_9_ being the insulin sensitivity coefficient, *k*_10_ is the glucose concentration gradient at which insulin-dependent glucose transport rate reaches its half-maximum (Michaelis constant), $\left(Glu_{b}-Glu_{m}\right)$ is the glucose concentration gradient across the muscle cell membrane serving as the driving force for glucose transport, *k*_11_ is the maximum rate of exercise-induced glucose uptake (zero under resting conditions), and *k*_12_ is the Michaelis constant representing glucose concentration gradient at which exercise-induced glucose transport rate reaches its half-maximum.

#### GLUT2-mediated glucose transport in liver

Hepatic glucose transport is primarily facilitated by glucose transporter 2 (GLUT2), a high-capacity, bidirectional transporter expressed on hepatocyte membranes (Thorens and Mueckler, 2010). GLUT2 enables rapid equilibration of glucose across the hepatic plasma membrane and is not considered rate-limiting under physiological conditions (Gould and Holman, 1993). This property allows the liver to efficiently uptake glucose following meals when blood glucose levels are elevated and to release glucose into the bloodstream during fasting when blood glucose levels decline. The ability of GLUT2 to mediate glucose transport without the need for regulated translocation is essential for the liver’s central role in maintaining systemic glucose homeostasis (Thorens and Mueckler, 2010). This implies that the glucose concentration in hepatocytes can be considered equal to blood glucose concentration:


$$Glu_{l}=Glu_{b}$$

where *Glu_l_* is liver or hepatocyte glucose concentration and *Glu_b_* is blood glucose concentration.

### Glycogen metabolism

#### Glycogenesis and glycogenolysis in liver

Hepatic glycogen metabolism is regulated by two opposing enzymatic processes: glycogenesis (glycogen synthesis) and glycogenolysis (glycogen breakdown), which are tightly regulated by hormonal signals, insulin and glucagon, respectively.

Endogenous glucose production is generally considered the sum of hepatic glycogenolysis and gluconeogenesis. Gluconeogenesis was not represented as a separate flux in the present model because, over the postprandial and overnight (≤780-minute) window studied, glycogenolysis is the dominant contributor to endogenous glucose production, whereas gluconeogenesis becomes quantitatively more important during prolonged fasting beyond the timescale captured in this protocol.

Glycogenesis, the process by which glucose is converted into glycogen, involves a series of enzymatically catalyzed biochemical reactions (Cross et al., 1995; Saltiel and Kahn, 2001), and can therefore be quantitatively modeled by the Michaelis-Menten equation with liver glucose (*Glu_l_*) as the substrate which is equal to blood glucose (*Glu_b_*):


$$r_{2}=\frac{k_{13}Ins^{k_{15}}\;Glu_{b}}{k_{14}+\;Glu_{b}}$$

where *k*_13_ $Ins^{k_{15}}$ is the maximum reaction rate modulated by insulin concentration with *k*_13_ being the insulin sensitivity coefficient, *k*_14_ is the Michaelis constant corresponding to the glucose concentration at which the reaction rate reaches one-half of its maximum, and exponent *k*_15_ is used to capture the nonlinear effect of insulin on glycogen synthase activity, which is modulated indirectly through a phosphorylation–dephosphorylation cascade. When insulin binds to its receptor, it activates the PI3K/Akt signaling pathway, leading to inhibition of glycogen synthase kinase-3 (GSK-3). Since GSK-3 inactivates glycogen synthase (GS) by phosphorylation, its inhibition promotes the dephosphorylated, active form of GS (Exton, 1979). This regulatory cascade introduces a nonlinear relationship between insulin concentration and glycogen synthesis rate, thereby entailing the use of the exponent *k*_15_.

Similarly, glycogenolysis in the liver is primarily stimulated by glucagon and can be represented in a Michaelis-Menten form with liver glycogen (*Gly_l_*) as substrate:


$$r_{3}=\frac{k_{16}Gcg^{k_{18}}\;\left(Gly_{l}+\frac{k_{19}}{Gly_{l}}\right)}{k_{17}+Gly_{l}}$$

where *k*_16_ $Gcg^{k_{18}}$ represents the maximum reaction rate modulated by glucagon concentration with *k*_16_ being the glucagon sensitivity coefficient, and *k*_17_ corresponds to the glycogen concentration at which the reaction rate reaches one-half of its maximum. $Gcg^{k_{18}}$ reflects the regulatory effect of glucagon on glycogen phosphorylase activity, the key enzyme responsible for catalyzing the release of glucose-1-phosphate from glycogen chains. Upon glucagon binding to its receptor on hepatocytes, adenylate cyclase is activated, leading to an increase in intracellular cyclic AMP (cAMP). Elevated cAMP levels activate protein kinase A (PKA), which in turn triggers a signaling cascade that results in the phosphorylation and activation of glycogen phosphorylase (Meléndez et al., 1999; Roach et al., 2012). The exponent *k*_18_ accounts for the nonlinear behavior of this glucagon-mediated signaling pathway.

In addition to substrate *Gly_l_*, a second term, $\frac{k_{19}}{Gly_{l}}$, is included to capture the structural effects of glycogen particles. In the liver, glycogen is stored as spherical β-particles (individual granules) and larger α-particles, which are aggregates of β-particles interconnected by protein linkers (Meléndez et al., 1999; Besford et al., 2016). Under normal physiological conditions, a balance between these particle types supports both efficient storage and mobilization of glucose. However, recent studies have shown that in diabetic mouse models, there is an increased proportion of α-particles relative to β-particles, suggesting a shift toward larger, more aggregated glycogen structures (Deng et al., 2015). These larger α-particles may be more resistant to enzymatic degradation, potentially impairing glycogen mobilization and contributing to hepatic glucose dysregulation in diabetes. Furthermore, the α-particles in diabetic livers have been found to be structurally fragile, readily disintegrating into β-particles under certain conditions (Nielsen and Richter, 2003; Deng et al., 2015). As glycogen is progressively depleted, α-particles fragment into smaller β-particles, increasing the access to reaction sites by glycogen phosphorylase, the rate-limiting enzyme in glycogenolysis. This effect is represented by $\frac{k_{19}}{Gly_{l}}$, a feedback mechanism that accelerates glycogen breakdown as glycogen stores diminish.

#### Glycogenesis and glycogenolysis in muscle

In skeletal muscle, absorbed glucose (as described by *r*_1_) is enzymatically converted into glycogen under the regulation by insulin, and can thus be modeled as:


$$r_{4}=\frac{k_{20}\frac{Ins^{k_{15}}}{Gly_{m}}\;Glu_{m}}{k_{21}+\;Glu_{m}}$$

where $k_{20}\frac{Ins^{k_{15}}}{Gly_{m}}$ is the maximum reaction rate and k_21_ denotes the intracellular glucose concentration at which the reaction rate reaches one-half of its maximum. $Ins^{k_{15}}$ represents the nonlinear regulatory effect of insulin on glycogen synthase activity. $\frac{1}{Gly_{m}}\;$ captures the enhancement of glycogen synthesis when muscle glycogen stores are depleted. This phenomenon has been well documented with studies showing that glycogen-depleted muscles exhibit enhanced insulin sensitivity and an accelerated rate of glycogen re-synthesis following exercise or fasting (Hawley et al., 1998; Jensen and Richter, 2012).

Glycogenolysis in skeletal muscle is predominantly driven by energy demands, particularly during physical activity (Richter and Hargreaves, 2013). Unlike hepatic glycogenolysis, which is hormonally regulated by glucagon, muscle glycogen breakdown is not directly affected by glucagon (Wojtaszewski et al., 2003). Instead, it is tightly linked to the intensity and duration of exercise (Romijn et al., 1993). During exercise, an increase in AMP levels, elevated intracellular calcium, and the activation of glycogen phosphorylase through phosphorylation by phosphorylase kinase lead to the rapid breakdown of glycogen stores, providing glucose-6-phosphate for ATP production to meet the energy demands of muscle contraction (Richter and Hargreaves, 2013; Ha et al., 2021).

### Kinetic model structure

The rates can be used to give the time rate of change or time derivatives ( $\frac{dx}{dt}$ with *x* being *Glu_b_* etc.) of the six state variables: Blood insulin concentration (*Ins*), Blood glucagon concentration (*Gcg*), Blood glucose concentration (*Glu_b_*), Muscle glucose concentration (*Glu_m_*), Muscle glycogen concentration (*Gly_m_*), and Liver glycogen concentration (*Gly_l_*). This gives six differential equations (Equations 1-6) as a kinetic model structure:

(1) $$\frac{dIns}{dt}=r_{Ins}^{in}-r_{Ins}^{out}$$

(2) $$\frac{dGcg}{dt}=r_{Gcg}^{in}-r_{Gcg}^{out}$$

(3) $$\frac{dGlu_{b}}{dt}=r_{infu}+r_{meal}+r_{3}-r_{2}-r_{1}-r_{bas}$$

(4) $$\frac{dGlu_{m}}{dt}=r_{1}-r_{4}$$

(5) $$\frac{dGly_{m}}{dt}=r_{4}-\;r_{exe}$$

(6) $$\frac{dGly_{l}}{dt}=r_{2}-\;r_{3}$$

In Equations 1, 2, $r_{Ins}^{in}$ and $r_{Ins}^{out}$ represent insulin secretion and clearance rates, respectively, while $r_{Gcg}^{in}$ and $r_{Gcg}^{out}$ represent glucagon secretion and clearance rates, respectively. Blood glucose variations (Equation 3) are determined by glucose infusion (*r_infu_*), postprandial glucose absorption (*r_meal_*), hepatic glycogenolysis (*r*_3_), hepatic glycogenesis (*r*_2_), skeletal muscle glucose uptake (*r*_1_), and basal glucose consumption (*r_bas_*). The basal glucose consumption term represents constitutive glucose utilization by tissues independent of insulin-mediated uptake, including glucose consumption by the brain and other tissues with relatively constant glucose demand. Muscle glucose (Equation 4) is increased by skeletal muscle glucose uptake (*r*_1_) and decreased through conversion to muscle glycogen (*r*_4_). Muscle glycogen (Equation 5) is formed through glycogenesis (*r*_4_) and consumed by exercise (*r_exe_*). Liver glycogen (Equation 6) is increased by hepatic glycogenesis (*r*_2_) and decreased by hepatic glycogenolysis (*r*_3_).

The rate equations are defined as follows (Equations 7-15):

(7) $$r_{Ins}^{in}=\frac{k_{1}Glu_{b}^{k_{3}}}{k_{2}^{k_{3}}+Glu_{b}^{k_{3}}}$$

(8) $$r_{Ins}^{out}=k_{4}Ins$$

(9) $$r_{Gcg}^{in}=\frac{k_{5}k_{6}^{k_{7}}}{k_{6}^{k_{7}}+Glu_{b}^{k_{7}}}$$

(10) $$r_{Gcg}^{out}=k_{8}Gcg$$

(11) $$r_{1}=\frac{k_{9}Ins\left(\;Glu_{b}-Glu_{m}\right)}{k_{10}+\left(Glu_{b}-Glu_{m}\right)}+\frac{k_{11}\left(\;Glu_{b}-Glu_{m}\right)}{k_{12}+\left(Glu_{b}-Glu_{m}\right)}$$

(12) $$r_{2}=\frac{k_{13}Ins^{k_{15}}\;Glu_{b}}{k_{14}+\;Glu_{b}}$$

(13) $$r_{3}=\frac{k_{16}Gcg^{k_{18}}\;\left(Gly_{l}+\frac{k_{19}}{Gly_{l}}\right)}{k_{17}+Gly_{l}}$$

(14) $$r_{4}=\frac{k_{20}\frac{Ins^{k_{15}}}{Gly_{m}}\;Glu_{m}}{k_{21}+\;Glu_{m}}$$

The postprandial glucose absorption rate (*r_meal_*) is modeled by a time-dependent function (Ha and Sherman, 2020):

(15) $$r_{meal}={\left(\frac{k_{22}\;t^{k23}}{k_{24}^{k_{23}}+t^{k23}}\right)}^{k_{25}}e^{-k_{26}t}$$

where *k*_22_ indicates the maximal rate of glucose absorption, *k*_23_ controls the sharpness of the rise in absorption, *k*_24_ determines the time (in minutes) required to reach half-maximal absorption, *k*_25_ shapes the overall absorption curve, and the exponential term $e^{-k_{26}t}$ accounts for the gradual decay in glucose absorption as the available glucose in the gastrointestinal tract diminishes over time.

### Parameter estimation and statistical analysis

Model parameters were estimated by optimizing model outputs against experimental measurements. The system of nonlinear differential equations was solved by using the fourth-order Runge-Kutta (RK4) method (Esfandiari, 2017). Parameter optimization was performed by using the Levenberg-Marquardt algorithm (Levenberg, 1944), which minimizes a weighted sum of squared differences between measured and predicted glucose, insulin, and glucagon concentrations over the experimental period. Specifically, the objective function was:


$$SSE=\sum_{j=1}^{M}w_{j}\sum_{i=1}^{N}{\left(y_{ij}^{exp}-y_{ij}^{model}\left(\theta \right)\right)}^{2}$$

where *θ* is the vector of model parameters, *N* is the number of experimental time points, *M* is the number of measured output variables (glucose, insulin, and glucagon), *w*_j_ is the weighting factor for variable *j*, $y_{ij}^{exp}$ and $y_{ij}^{model}$ denote the experimental and model-predicted values, respectively. To account for the differences in numerical magnitudes of the output variables, the errors were normalized with fixed *w*_j_ based on the maximum of measured insulin, glucagon, or glucose. The damping factor in parameter optimization was varied as $\lambda \text{diag }\left(J^{T}J\right)$, where λ being constant and *J* being the Jacobian matrix. Initial parameter values were selected from published physiological estimates and refined through preliminary simulations before optimization. Optimization was considered converged when changes the objective function between successive iterations were below a threshold value. After convergence, the coefficient of variation (CV) for an estimated parameter was computed as $100\text{diag}{\left(\sigma^{2}{\left(J^{T}J\right)}^{-1}\right)}^{1/2}/|\hat{\theta }|$ with σ^2^ being the residual variance and $\hat{\theta }$ being the estimated parameter value.

Statistical analyses were limited to prespecified comparisons of the main effects. Group comparisons under the no-exercise condition (**Table 1**) were performed by using one-way ANOVA followed by Fisher’s Least Significant Difference (LSD) test for predefined pairwise comparisons. For the exercise intervention (**Tables 2**–**4**), because each participant completed all three exercise conditions (NOEX, AMEX, and PMEX), repeated-measures ANOVA followed by Fisher’s LSD test was used to compare exercise conditions within each metabolic group. Statistical significance was defined as *P* < 0.05. Because multiple model parameters were evaluated, these statistical comparisons were considered exploratory, and nominal *P*-values were not adjusted for multiple comparisons. Accordingly, findings from these comparisons should be interpreted as hypothesis-generating rather than confirmatory. Coefficient of determination (R^2^) was computed to measure the agreement between model predictions and experiments. All the programming and analysis were carried out in MATLAB (The MathWorks, Natick, MA, USA).

**Table 1:** Definitions of model parameters and estimated values (average ± SD).

Symbol	Parameter definition	Unit	Obese (n=18) NOEX		Diabetic (n=16) NOEX		Non-Obese (n=18) NOEX	
			parameter (mean ± std)	CV (%) [min, max]	parameter (mean ± std)	CV (%) [min, max]	parameter (mean ± std)	CV (%) [min, max]
*k*_1_	Maximum rate of insulin secretion	nmol/L/min	0.0501 ± 0.0530	[8.19, 54.02]	0.0261 ± 0.0311	[0, 37.40]	0.0521 ± 0.0596	[1.10, 89.53]
*k*_2_	Glucose concentration at half-maximal insulin secretion	nmol/L/min	7.1489 ± 1.0896	[4.40, 28.73]	8.0261 ± 1.1581	[0, 47.93]	7.3664 ± 0.9043	[1.63, 27.11]
*k_3_*	Cooperativity of glucose-stimulated insulin secretion (Hill coefficient)	–	9.9788 ± 0.7598	[5.56, 71.16]	9.9857 ± 1.0090	[0, 170.51]	10.1272 ± 0.7999	[0.57, 43.41]
*k_4_*	Insulin clearance rate	1/min	0.0147 ± 0.0036	[5.56, 26.57]	0.0126 ± 0.0089	[3.12, 43.13]	0.0127 ± 0.0024	[0.97, 50.00]
*k*_5_	Maximum rate of glucagon secretion	pmol/L/min	3.3816 ± 1.5874	[0, 41.91]	4.4566 ± 2.0219	[0, 42.08]	4.1279 ± 0.5931	[3.07, 15.13]
*k*_6_	Glucose concentration at half-maximal glucagon secretion	pmol/L	2.0945 ± 0.2668	[3.49, 26.87]	2.0466 ± 0.5266	[0.15, 25.74]	2.1181 ± 0.7197	[1.28, 35.81]
*k*_7_	Cooperativity of glucose-inhibited glucagon secretion (Hill coefficient)	–	4.3650 ± 1.1622	[5.56, 66.36]	3.4670 ± 1.3855	[0, 36.19]	4.4489 ± 1.8034	[1.40, 38.32]
*k*_8_	Glucagon clearance rate	1/min	0.0100 ± 0.0097	[7.89, 65.02]	0.0173 ± 0.0152	[4.00, 37.92]	0.0223 ± 0.0227**^#^**	[4.34, 34.94]
*k*_9_	Insulin sensitivity coefficient for muscle glucose uptake	–	2.2793 ± 0.1227	[10.04, 170.03]	3.0498 ± 1.5051	[4.70, 48.94]	2.3186 ± 0.1061*	[2.44, 96.41]
*k*_10_	Glucose concentration at half-maximal insulin-stimulated uptake	mmol/L	3.0271 ± 0.0677	[80.16, 104.85]	3.1731 ± 1.3183	[0.07, 162.75]	3.0063 ± 0.0641	[2.54, 40.30]
*k*_11_	Maximal exercise-induced glucose uptake by muscle	mmol/min	0.0000 ± 0.0000	fixed	0.0000 ± 0.0000	fixed	0.0000 ± 0.0000	fixed
*k*_12_	Glucose concentration at half-maximal exercise-induced uptake	mmol/L	3.0271 ± 0.0677	[30.18, 76.54]	3.1731 ± 1.3183	[68.19, 126.38]	3.0063 ± 0.0641	[58.10, 156.33]
*k*_13_	Insulin sensitivity coefficient for hepatic glycogenesis	–	4.9289 ± 0.8088	[39.81, 54.14]	5.1340 ± 0.9853	[13.43, 89.84]	5.4059 ± 0.4966**^#^**	[20.90, 66.86]
*k*_14_	Glucose concentration at half-maximal hepatic glycogenesis	mmol/L	8.7580 ± 0.2085	[61.62, 93.04]	10.3967 ± 3.9155	[1.04, 171.81]	8.5574 ± 0.1294**^#^**	[12.61, 89.84]
*k*_15_	Nonlinear effect of insulin on glycogen synthase activity	–	0.8090 ± 0.2449	[0, 174.67]	0.9376 ± 0.5739	[50.50, 97.38]	0.8383 ± 0.2948	[6.67, 122.24]
*k*_16_	Glucagon sensitivity coefficient for hepatic glycogenolysis	–	0.1783 ± 0.1170	[4.53, 44.14]	0.4010 ± 0.4791	[10.97, 73.95]	0.1684± 0.0558*	[8.06, 51.24]
*k*_17_	Hepatic glycogen concentration at half-maximal glycogenolysis	mmol/L	0.0011 ± 0.0020	[75.94, 189.37]	0.0145 ± 0.0435	[3.09, 111.15]	0.0010 ± 0.0017	[99.13, 159.50]
*k*_18_	Nonlinear effect of glucagon on glycogen phosphorylase activity	–	0.4852 ± 0.0894	[67.52, 125.11]	0.5414 ± 0.3395	[10.18, 51.72]	0.4724 ± 0.0826	[36.87, 76.49]
*k*_19_	Feedback effect of glycogen store on hepatic glycogenolysis	–	0.0004 ± 0.0014	[2.62, 50.30]	10.8580 ± 19.8479	[15.49, 169.73]	0.0004± 0.0010*	[49.00, 94.31]
*k*_20_	Insulin sensitivity coefficient for glycogenesis in muscle	–	5.9381 ± 0.4074	[1.53, 87.39]	5.4993 ± 2.1565	[35.80, 49.21]	6.1712± 0.0835*	[59.42, 80.60]
*k*_21_	Muscle glucose concentration at half-maximal glycogenesis	mmol/L	0.1597 ± 0.3805	[2.19, 37.00]	1.6257 ± 2.4585	[23.25, 41.64]	0.0509± 0.2032*	[20.98, 118.21]
*k*_22_	Maximum glucose absorption rate from meal	mmol/min	8.2764 ± 0.0000	fixed	8.2764 ± 0.0000	fixed	8.2764 ± 0.0000	fixed
*k*_23_	Steepness of glucose absorption rise (sharpness factor)	–	4.0000 ± 0.0000	fixed	4.0000 ± 0.0000	fixed	4.0000 ± 0.0000	fixed
*k*_24_	Time to reach half-maximal meal glucose absorption	min	40.0000 ± 0.0000	fixed	40.0000 ± 0.0000	fixed	40.0000 ± 0.0000	fixed
*k*_25_	Shape parameter of meal absorption curve	–	0.3000 ± 0.0000	fixed	0.3000 ± 0.0000	fixed	0.3000 ± 0.0000	fixed
*k*_26_	Rate constant for postprandial meal absorption decline	1/min	0.0150 ± 0.0000	fixed	0.0150 ± 0.0000	fixed	0.0150 ± 0.0000	fixed

**^#^**Denotes statistically significant difference (p< 0.05) between obese and non-obese groups.

*Denotes statistically significant difference (p< 0.05) between diabetic and non-obese groups.

**Table 2:** Model parameter definitions and corresponding estimated values for the non-obese group across morning exercise (AMEX), post-meal exercise (PMEX), and no exercise (NOEX) conditions (mean ± SD).

Symbol	Parameter definition	Unit	Non-obese (AMEX)	Non-obese (PMEX)	Non-obese (NOEX)
*k*_1_	Maximum rate of insulin secretion	nmol/L/min	0.8650 ± 3.0766	0.8461 ± 3.2386	0.0521 ± 0.0596
*k*_2_	Glucose concentration at half-maximal insulin secretion	nmol/L/min	8.0883 ± 4.1619	8.3269 ± 3.0512	7.3664 ± 0.9043
*k_3_*	Cooperativity of glucose-stimulated insulin secretion (Hill coefficient)	–	8.0000 ± 0.0000	8.0000 ± 0.0000	10.1272 ± 0.7999
*k_4_*	Insulin clearance rate	1/min	0.0140 ± 0.0054	0.0141 ± 0.0040	0.0127 ± 0.0024
*k*_5_	Maximum rate of glucagon secretion	pmol/L/min	6.9639 ± 6.5246	6.3913 ± 5.8126	4.1279 ± 0.5931
*k*_6_	Glucose concentration at half-maximal glucagon secretion	pmol/L	1.9829 ± 0.9372	3.1050 ± 4.7754	2.1181 ± 0.7197
*k*_7_	Cooperativity of glucose-inhibited glucagon secretion (Hill coefficient)	–	5.5175 ± 2.7813	5.0986 ± 2.9892	4.4489 ± 1.8034
*k*_8_	Glucagon clearance rate	1/min	0.0114 ± 0.0104	0.0685 ± 0.2350	0.0223 ± 0.0227
*k*_9_	Insulin sensitivity coefficient for muscle glucose uptake	–	3.4237 ± 3.2589	2.8928 ± 3.2123	2.3186 ± 0.1061
*k*_10_	Glucose concentration at half-maximal insulin-stimulated uptake	mmol/L	2.9469 ± 4.2586	2.6490 ± 4.8029	3.0063 ± 0.0641
*k*_11_	Maximal exercise-induced glucose uptake by muscle	mmol/min	0.0000 ± 0.0000	0.0000 ± 0.0000	0.0000 ± 0.0000
*k*_12_	Glucose concentration at half-maximal exercise-induced uptake	mmol/L	2.9469 ± 4.2586	2.6490 ± 4.8029	3.0063 ± 0.0641
*k*_13_	Insulin sensitivity coefficient for hepatic glycogenesis	–	21.7457 ± 38.4632	9.0363 ± 6.5990	5.4059 ± 0.4966*
*k*_14_	Glucose concentration at half-maximal hepatic glycogenesis	mmol/L	19.3316 ± 34.3084	5.4737 ± 9.0460	8.5574 ± 0.1294
*k*_15_	Nonlinear effect of insulin on glycogen synthase activity	–	1.0000 ± 0.0000	1.0000 ± 0.0000	0.8383 ± 0.2948
*k*_16_	Glucagon sensitivity coefficient for hepatic glycogenolysis	–	0.1113 ± 0.1252	0.1021 ± 0.0626	0.1684 ± 0.0558*
*k*_17_	Hepatic glycogen concentration at half-maximal glycogenolysis	mmol/L	0.7161 ± 2.2651	0.0708 ± 0.2889	0.0010 ± 0.0017
*k*_18_	Nonlinear effect of glucagon on glycogen phosphorylase activity	–	0.4724 ± 0.0826	0.4724 ± 0.0826	0.4724 ± 0.0826
*k*_19_	Feedback effect of glycogen store on hepatic glycogenolysis	–	27.4833 ± 26.3771	15.8030 ± 16.8699	0.0004 ± 0.0010^#^*
*k*_20_	Insulin sensitivity coefficient for glycogenesis in muscle	–	22.2856 ± 22.3096	19.7227 ± 17.6685	6.1712 ± 0.0835^#^*
*k*_21_	Muscle glucose concentration at half-maximal glycogenesis	mmol/L	8.8035 ± 25.6928	2.3526 ± 5.3287	0.0509 ± 0.2032
*k*_22_	Maximum glucose absorption rate from meal	mmol/min	8.2764 ± 0.0000	8.2764 ± 0.0000	8.2764 ± 0.0000
*k*_23_	Steepness of glucose absorption rise (sharpness factor)	–	4.0000 ± 0.0000	4.0000 ± 0.0000	4.0000 ± 0.0000
*k*_24_	Time to reach half-maximal meal glucose absorption	min	40.0000 ± 0.0000	40.0000 ± 0.0000	40.0000 ± 0.0000
*k*_25_	Shape parameter of meal absorption curve	–	0.3000 ± 0.0000	0.3000 ± 0.0000	0.3000 ± 0.0000
*k*_26_	Rate constant for postprandial meal absorption decline	1/min	0.0150 ± 0.0000	0.0150 ± 0.0000	0.0150 ± 0.0000

**^#^**Denotes a statistically significant difference (p< 0.05) between AMEX and NOEX.

*Denotes a statistically significant difference (p< 0.05) between PMEX and NOEX.

**Table 3:** Model parameter definitions and corresponding estimated values for the obese group across morning exercise (AMEX), post-meal exercise (PMEX), and no exercise (NOEX) conditions (mean ± SD).

Symbol	Parameter definition	Unit	Obese (AMEX)	Obese (PMEX)	Obese (NOEX)
*k*_1_	Maximum rate of insulin secretion	nmol/L/min	0.0885 ± 0.1400	0.1082 ± 0.2350	0.0482 ± 0.0521
*k*_2_	Glucose concentration at half-maximal insulin secretion	nmol/L/min	7.4363 ± 1.8741	7.7771 ± 1.9249	7.1533 ± 1.0572
*k_3_*	Cooperativity of glucose-stimulated insulin secretion (Hill coefficient)	–	8.0427 ± 0.1811	8.0427 ± 0.1811	9.9115 ± 0.7904
*k_4_*	Insulin clearance rate	1/min	0.0182 ± 0.0099	0.0202 ± 0.0169	0.0145 ± 0.0037
*k*_5_	Maximum rate of glucagon secretion	pmol/L/min	7.4890 ± 6.2134	6.8498 ± 6.1517	3.8371 ± 2.4711^#^*
*k*_6_	Glucose concentration at half-maximal glucagon secretion	pmol/L	1.8127 ± 0.9209	1.9495 ± 0.8931	2.1117 ± 0.2689
*k*_7_	Cooperativity of glucose-inhibited glucagon secretion (Hill coefficient)	–	5.0079 ± 2.6922	5.3247 ± 2.8207	4.4809 ± 1.2300
*k*_8_	Glucagon clearance rate	1/min	0.0095 ± 0.0114	0.0138 ± 0.0129	0.0103 ± 0.0095
*k*_9_	Insulin sensitivity coefficient for muscle glucose uptake	–	2.3727 ± 2.7405	2.6864 ± 2.7272	2.4632 ± 0.7893
*k*_10_	Glucose concentration at half-maximal insulin-stimulated uptake	mmol/L	3.5156 ± 5.0707	3.1773 ± 5.2721	3.0287 ± 0.0660
*k*_11_	Maximal exercise-induced glucose uptake by muscle	mmol/min	0.0000 ± 0.0000	0.0000 ± 0.0000	0.0000 ± 0.0000
*k*_12_	Glucose concentration at half-maximal exercise-induced uptake	mmol/L	3.5156 ± 5.0707	3.1773 ± 5.2721	3.0287 ± 0.0660
*k*_13_	Insulin sensitivity coefficient for hepatic glycogenesis	–	9.7852 ± 8.6186	5.7306 ± 6.3131	4.9483 ± 0.7890^#^
*k*_14_	Glucose concentration at half-maximal hepatic glycogenesis	mmol/L	22.6129 ± 21.5102	7.6791 ± 10.1667	8.7529 ± 0.2034^#^
*k*_15_	Nonlinear effect of insulin on glycogen synthase activity	–	1.0054 ± 0.0230	1.0054 ± 0.0230	0.8250 ± 0.2471
*k*_16_	Glucagon sensitivity coefficient for hepatic glycogenolysis	–	0.0985 ± 0.0371	0.0947 ± 0.0390	0.1770 ± 0.1136^#^*
*k*_17_	Hepatic glycogen concentration at half-maximal glycogenolysis	mmol/L	0.2708 ± 0.7554	0.1760 ± 0.4150	0.0010 ± 0.0019
*k*_18_	Nonlinear effect of glucagon on glycogen phosphorylase activity	–	0.4873 ± 0.0872	0.4873 ± 0.0872	0.4873 ± 0.0872
*k*_19_	Feedback effect of glycogen store on hepatic glycogenolysis	–	9.1810 ± 11.8170	14.8965 ± 21.6065	0.0004 ± 0.0013^#^*
*k*_20_	Insulin sensitivity coefficient for glycogenesis in muscle	–	10.3799 ± 11.8451	15.5347 ± 11.0379	6.4555 ± 2.2303*
*k*_21_	Muscle glucose concentration at half-maximal glycogenesis	mmol/L	4.3053 ± 8.7767	2.6121 ± 4.9613	0.1508 ± 0.3711
*k*_22_	Maximum glucose absorption rate from meal	mmol/min	9.1377 ± 3.6542	9.1377 ± 3.6542	9.1377 ± 3.6542
*k*_23_	Steepness of glucose absorption rise (sharpness factor)	–	4.0000 ± 0.0000	4.0000 ± 0.0000	4.0000 ± 0.0000
*k*_24_	Time to reach half-maximal meal glucose absorption	min	43.3333 ± 14.1421	43.3333 ± 14.1421	43.3333 ± 14.1421
*k*_25_	Shape parameter of meal absorption curve	–	0.3111 ± 0.0471	0.3111 ± 0.0471	0.3111 ± 0.0471
*k*_26_	Rate constant for postprandial meal absorption decline	1/min	0.0150 ± 0.0000	0.0150 ± 0.0000	0.0150 ± 0.0000

**^#^**Denotes a statistically significant difference (p< 0.05) between AMEX and NOEX.

*Denotes a statistically significant difference (p< 0.05) between PMEX and NOEX.

**Table 4:** Model parameter definitions and corresponding estimated values for the diabetic group across morning exercise (AMEX), post-meal exercise (PMEX), and no exercise (NOEX) conditions (mean ± SD).

Symbol	Parameter definition	Unit	Diabetic (AMEX)	Diabetic (PMEX)	Diabetic (NOEX)
*k*_1_	Maximum rate of insulin secretion	nmol/L/min	0.0260 ± 0.0310	0.0601 ± 0.1436	0.0261 ± 0.0311
*k*_2_	Glucose concentration at half-maximal insulin secretion	nmol/L/min	7.9582 ± 1.4885	8.8581 ± 2.7454	8.0261 ± 1.1581
*k_3_*	Cooperativity of glucose-stimulated insulin secretion (Hill coefficient)	–	8.0000 ± 0.0000	8.0000 ± 0.0000	9.9857 ± 1.0090
*k_4_*	Insulin clearance rate	1/min	0.0173 ± 0.0143	0.0125 ± 0.0077	0.0126 ± 0.0089
*k*_5_	Maximum rate of glucagon secretion	pmol/L/min	4.4440 ± 3.2982	7.4136 ± 4.0889	4.4566 ± 2.0219
*k*_6_	Glucose concentration at half-maximal glucagon secretion	pmol/L	3.3815 ± 5.1638	2.5319 ± 1.7989	2.0466 ± 0.5266
*k*_7_	Cooperativity of glucose-inhibited glucagon secretion (Hill coefficient)	–	4.0545 ± 2.8483	5.2017 ± 3.1303	3.4670 ± 1.3855
*k*_8_	Glucagon clearance rate	1/min	0.0137 ± 0.0154	0.0141 ± 0.0154	0.0173 ± 0.0152
*k*_9_	Insulin sensitivity coefficient for muscle glucose uptake	–	4.0749 ± 6.7701	2.7485 ± 2.7911	3.0498 ± 1.5051
*k*_10_	Glucose concentration at half-maximal insulin-stimulated uptake	mmol/L	2.0216 ± 2.9164	1.9403 ± 3.4284	3.1731 ± 1.3183
*k*_11_	Maximal exercise-induced glucose uptake by muscle	mmol/min	0.0000 ± 0.0000	0.0000 ± 0.0000	0.0000 ± 0.0000
*k*_12_	Glucose concentration at half-maximal exercise-induced uptake	mmol/L	2.0216 ± 2.9164	1.9403 ± 3.4284	3.1731 ± 1.3183
*k*_13_	Insulin sensitivity coefficient for hepatic glycogenesis	–	10.3669 ± 11.9036	6.9349 ± 4.4591	5.1340 ± 0.9853
*k*_14_	Glucose concentration at half-maximal hepatic glycogenesis	mmol/L	22.4653 ± 17.4645	16.9236 ± 15.6358	10.3967 ± 3.9155^#^
*k*_15_	Nonlinear effect of insulin on glycogen synthase activity	–	1.0000 ± 0.0000	1.0000 ± 0.0000	0.9376 ± 0.5739
*k*_16_	Glucagon sensitivity coefficient for hepatic glycogenolysis	–	0.2089 ± 0.1789	0.1738 ± 0.1628	0.4010 ± 0.4791^#^*
*k*_17_	Hepatic glycogen concentration at half-maximal glycogenolysis	mmol/L	4.9147 ± 13.7428	1.1797 ± 2.4728	0.0145 ± 0.0435
*k*_18_	Nonlinear effect of glucagon on glycogen phosphorylase activity	–	0.5539 ± 0.3194	0.5539 ± 0.3194	0.5414 ± 0.3395
*k*_19_	Feedback effect of glycogen store on hepatic glycogenolysis	–	14.0311 ± 19.1692	21.0997 ± 20.7462	10.8580 ± 19.8479
*k*_20_	Insulin sensitivity coefficient for glycogenesis in muscle	–	12.6126 ± 21.8955	7.3341 ± 6.3822	5.4993 ± 2.1565
*k*_21_	Muscle glucose concentration at half-maximal glycogenesis	mmol/L	5.1623 ± 9.4323	4.2116 ± 6.0579	1.6257 ± 2.4585
*k*_22_	Maximum glucose absorption rate from meal	mmol/min	8.2764 ± 0.0000	8.2764 ± 0.0000	8.2764 ± 0.0000
*k*_23_	Steepness of glucose absorption rise (sharpness factor)	–	4.0000 ± 0.0000	4.0000 ± 0.0000	4.0000 ± 0.0000
*k*_24_	Time to reach half-maximal meal glucose absorption	min	40.0000 ± 0.0000	40.0000 ± 0.0000	40.0000 ± 0.0000
*k*_25_	Shape parameter of meal absorption curve	–	0.3000 ± 0.0000	0.3000 ± 0.0000	0.3000 ± 0.0000
*k*_26_	Rate constant for postprandial meal absorption decline	1/min	0.0150 ± 0.0000	0.0150 ± 0.0000	0.0150 ± 0.0000

**^#^**Denotes a statistically significant difference (p< 0.05) between AMEX and NOEX.

*Denotes a statistically significant difference (p< 0.05) between PMEX and NOEX.

### Initial conditions

Initial blood plasma concentrations of insulin, glucagon, and glucose were set to the first measured values for each participant. Muscle glucose concentration was assumed to be initially equal to the blood glucose concentration.

Baseline glycogen concentrations in muscle and liver were based on physiological estimates in the literature. Muscle glycogen content was set to 100 mmol/L, consistent with resting values typically observed in non-obese individuals (Jensen and Richter, 2012). Resting muscle glycogen concentrations generally range from 80 to 120 mmol/L, depending on factors such as fitness level, diet, and recent physical activity.

For liver glycogen, an initial concentration of 250 mmol/L (expressed in unit of glucose) was assumed according to reported values for the fed state in humans (Taylor et al., 1996). Liver glycogen stores are dynamic and can vary between approximately 200 to 400 mmol/L depending on the time since the last meal, overall metabolic status, and hormonal regulation (Taylor et al., 1996).

## Results

### Model structure validation

The model structure (**Figure 1**, Equations 1-6) is based on the underlying physical or biochemical processes. Consequently, the model parameters are physically meaningful process coefficients (see **Table 1**) that define a system and would thus vary from person to person. If the model can describe measured outputs from different individuals (insulin, glucagon, and glucose over 13 hours from 52 individuals in three metabolic health conditions in this study) by varying only the coefficients, then the model is *structurally valid*, meaning that its structure can capture *the forms* of the underlying kinetics. Determining the model parameters by minimizing errors between model outputs and measured values is solving the *inverse problem* of finding model coefficients from measurable variables, commonly referred to as soft sensing or system identification. In doing so, the model structure is used as a filter to convert measurements into coefficient values. Variations in the model coefficients represent differences among individuals and can thus be analyzed to observe such differences, as discussed in later sections. This is different from attempts to develop an empirical predictive model (by using regression or neural networks, for example) where a set of unified parameter values is sought through a training and validation process.

**Figure 2:**
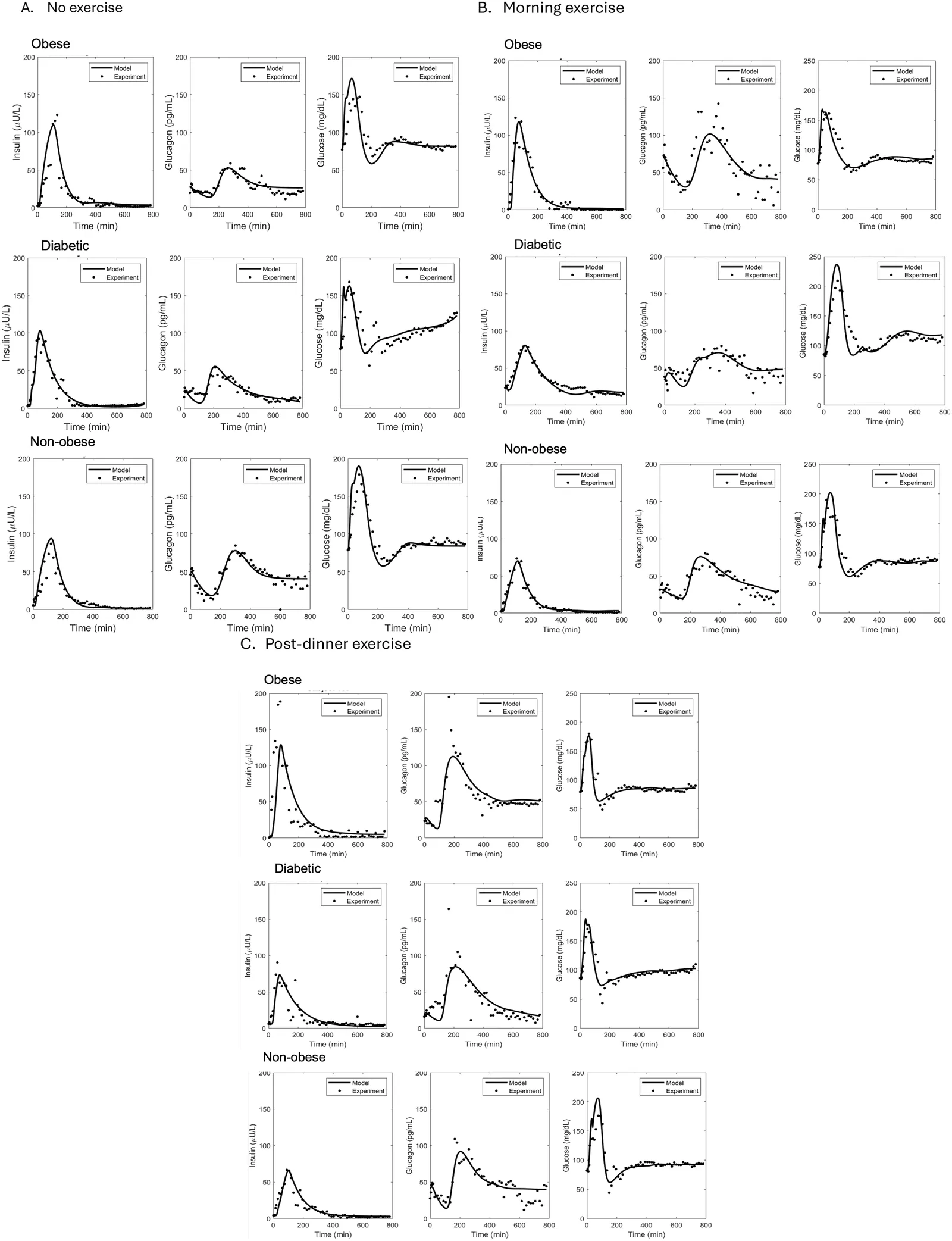
Example plots comparing model predictions against experimental measurements: **(A)** NOEX: no exercise, **(B)** AMEX: morning exercise, and **(C)** PMEX: post-dinner exercise. Lines represent model predictions with subject-specific optimized parameters over 780 minutes post-meal; dots are experimental measurements.

Comparisons between model predictions and experimental measurements of insulin, glucagon, and blood glucose variations for a representative subject from each group (non-obese, obese, and diabetic) are shown in **Figure 2**. By varying only the parameter values, the model structure can closely reproduce the temporal profiles of insulin, glucagon, and blood glucose for the participants in the three groups, with coefficient of determination (R^2^) values of 0.85 ± 0.09 for insulin, 0.78 ± 0.08 for glucagon, and 0.80 ± 0.06 for glucose, respectively. Because model parameters were estimated independently for each participant, the reported R^2^ values measure the structural adequacy of the proposed mechanistic model rather than its predictive performance on unseen individuals. A strong agreement between simulated and measured insulin, glucagon, and glucose dynamics across participants demonstrates that the proposed model structure captures the dominant physiological processes governing glucose regulation over the experimental time course. The means and standard deviations of the optimized model parameter values obtained for each health condition are provided in **Table 1**.

The coefficients of variation (CV) for the estimated parameters are reported in **Table 1** as mean plus the minimum and the maximum values representing the range for all participants in each group. For a complex system with much participant-to-participant variations, the CV values indicate acceptable uncertainties or identifiability for most of the parameters, but several parameters showed elevated CV values for some groups. Most notably, the maximum CV for *k*_10_, *k*_16_, and *k*_17_ exceeded 100% for two groups. While this usually resulted from one or a few participants in a group, the values indicate substantial estimation uncertainties. This will be further discussed in the Limitations subsection.

**Figure 3:**
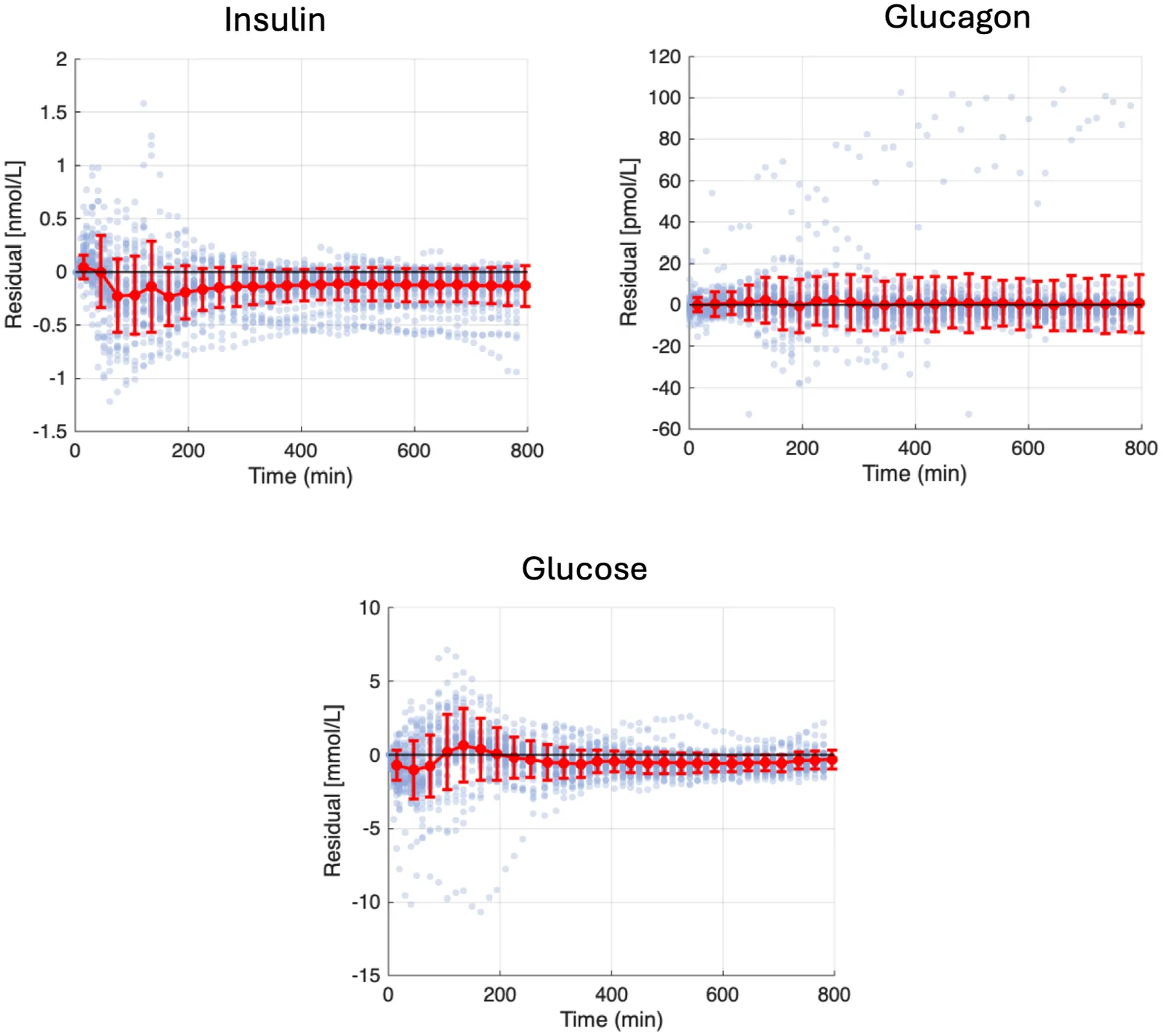
Residual analysis of model fits across all participants. Residuals are shown as a function of time for insulin, glucagon, and glucose across 52 participants, comprising 2,912 observation points for each variable. Blue dots represent residuals for individual observations. Red circles indicate the mean residual across participants at each time point, and red error bars indicate standard deviation. The horizontal black line denotes zero residual.

The residuals for insulin, glucagon, and glucose across all 52 participants are plotted as functions of time in **Figure 3**. The residuals generally hovered around zero but varied over time to a degree in terms of mean and variance, indicating limitations in the model as further discussed in the Limitations subsection. The root mean squared error (RMSE) was computed for each participant. Across the 52 participants, the mean ± SD RMSE was 0.2239 ± 0.1214 nmol/L for insulin, 6.4170 ± 19.7031 pmol/L for glucagon, and 1.2057 ± 0.6991 mmol/L for glucose. These values are consistent with the R^2^ values in indicating good agreement between model simulations and experimental measurements.

### Model coefficient values

As mentioned above, the model structure serves as a characterization of the system and the model parameters are process coefficients reflecting differences among individuals or groups. In comparison to non-obese controls, obese individuals exhibited multiple significant alterations in key glucose-regulatory parameters (**Table 1**). Notably, the glucagon clearance rate (*k*_8_​) was significantly lower in obese participants (0.0100 ± 0.0097 min^-1^) than in non-obese individuals (0.0223 ± 0.0227 min^-1^) (p< 0.05). Additionally, hepatic glucose storage capacity appeared impaired in obese individuals as exhibited by a significantly reduced insulin sensitivity coefficient for hepatic glycogen synthesis (*k*_13_​: 4.9289 ± 0.8088 vs. 5.4059 ± 0.4966 for the non-obese group, p< 0.05) and an elevated glucose concentration required for half-maximal hepatic glycogenesis (*k*_14_​: 8.7580 ± 0.2085 mM vs. 8.5574 ± 0.1294 mM for the non-obese group, p< 0.05). Furthermore, the insulin sensitivity coefficient for muscle glycogen synthesis (*k*_20_​) was significantly reduced in the obese group (5.9381 ± 0.4074) compared to non-obese individuals (6.1712 ± 0.0835) (p< 0.05).

The insulin sensitivity coefficient for muscle glucose uptake (*k*_9_​) was significantly higher in the diabetic group (3.0498 ± 1.5051) compared to non-obese controls (2.3186 ± 0.1061) (p< 0.05). Additionally, individuals with type 2 diabetes exhibited a markedly elevated glucagon sensitivity coefficient for hepatic glycogen breakdown (*k*_16_: 0.4010 ± 0.4791) and a significantly elevated *k*_19_ (10.8580 ± 19.8479) (p< 0.05) compared to non-obese individuals (0.1684 ± 0.0558) (p< 0.05). Lastly, the muscle glucose concentration required for half-maximal glycogen synthesis (*k*_21_​) was significantly higher in individuals with type 2 diabetes (1.6257 ± 2.4585 mM) compared to non-obese controls (0.0509 ± 0.2032 mM) (p< 0.05).

### Net hepatic glucose balance

**Figure 4:**
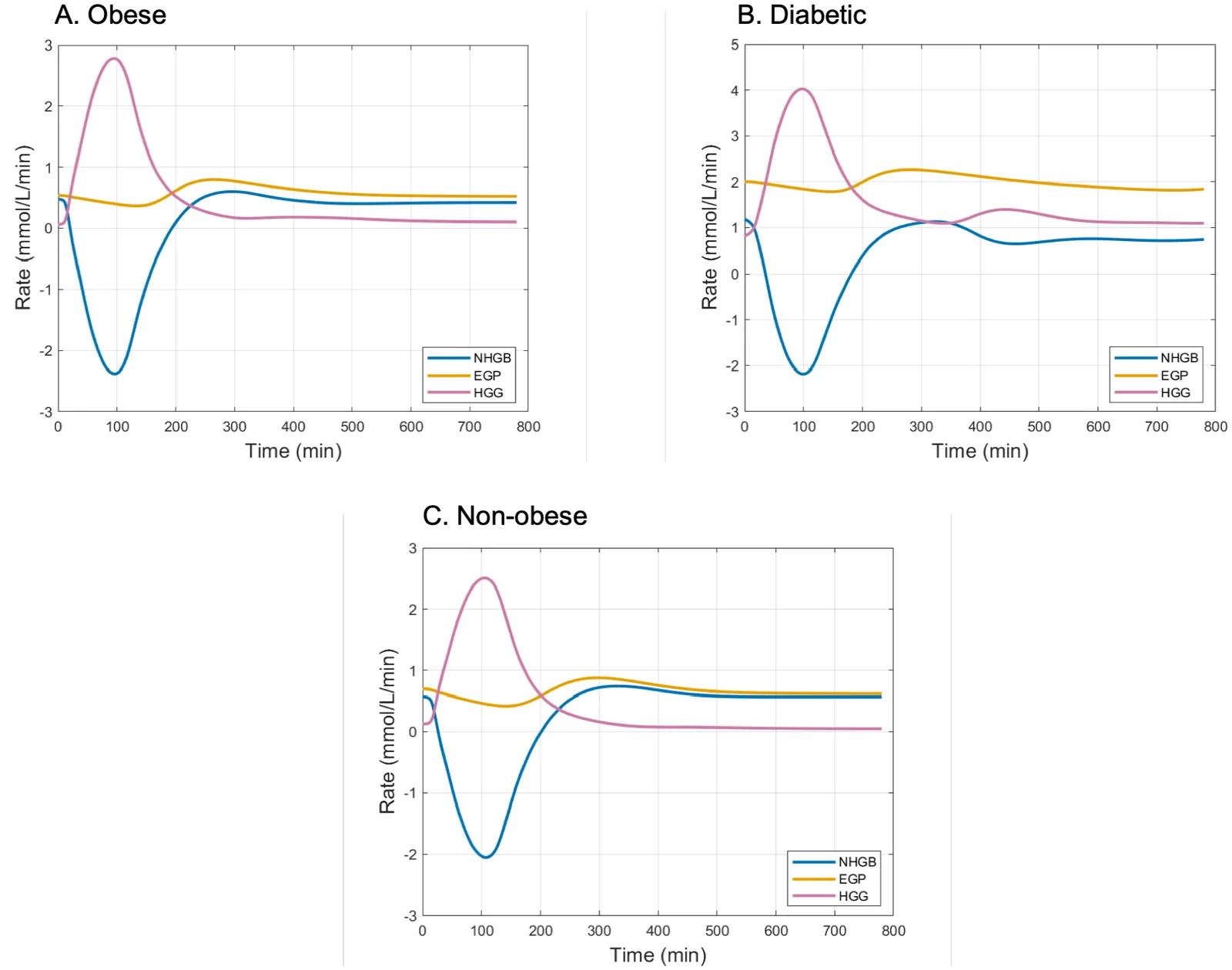
Representative simulated profiles of hepatic glucose dynamics in individuals with obesity **(A)**, diabetes **(B)**, and non-obese individuals **(C)**. Lines depict model-predicted trajectories over 780 minutes following a meal, generated with subject-specific optimized parameters. The blue line represents net hepatic glucose balance (NHGB), the yellow line indicates endogenous glucose production (EGP), and the pink line denotes hepatic glucogenesis (HGG). Key finding: Diabetic participants exhibited oscillatory and delayed recovery of NHGB during fasting, consistent with impaired hormonal coordination of hepatic glucose regulation, while non-obese and obese groups showed smooth recovery by approximately 300 minutes.

Using subject-specific optimized parameters, we simulated net hepatic glucose balance (NHGB), endogenous glucose production (EGP) and hepatic glucogenesis (HGG) profiles for obese, diabetic, and non-obese individuals (**Figures 4A–C**). NHGB represents the net hepatic glucose output, calculated as the difference between glycogenolysis (glucose release, EGP) and glycogenesis (glucose storage, HGG). This balance is tightly regulated by the interplay of insulin and glucagon signaling.

In the initial 100 minutes following meal ingestion, all three groups exhibited a sharp decline in NHGB, reaching a minimum near 100 minutes. This decline is predominantly driven by the postprandial rise in insulin (**Figure 2**), which strongly stimulates hepatic glycogen synthesis (HGG). During this period, glucagon levels remain low, resulting in minimal glycogenolysis (EGP). Consequently, the rate of glucose storage surpasses release, yielding a negative NHGB. From 100–200 minutes, insulin levels gradually decline, thereby reducing the stimulatory effect on glycogenesis. Glucagon remains relatively low but starts to rise (**Figure 2**). The NHGB begins to recover slightly toward baseline because of a reduction in glucose storage rather than an increase in glucose production. The balance remains negative, but the absolute magnitude of suppression diminishes. After 200 minutes, insulin concentrations fall to near-baseline levels, while glucagon action becomes increasingly dominant. For most diabetic participants, NHGB recovery exhibits oscillatory fluctuations (**Figure 4B**), reflecting impaired function of glucagon and dysregulated hepatic feedback mechanisms. While the non-obese and obese individuals show a smooth and timely return of NHGB to baseline by near 300 minutes, reflecting intact hormonal coordination and metabolic flexibility in hepatic glucose regulation.

**Figure 5:**
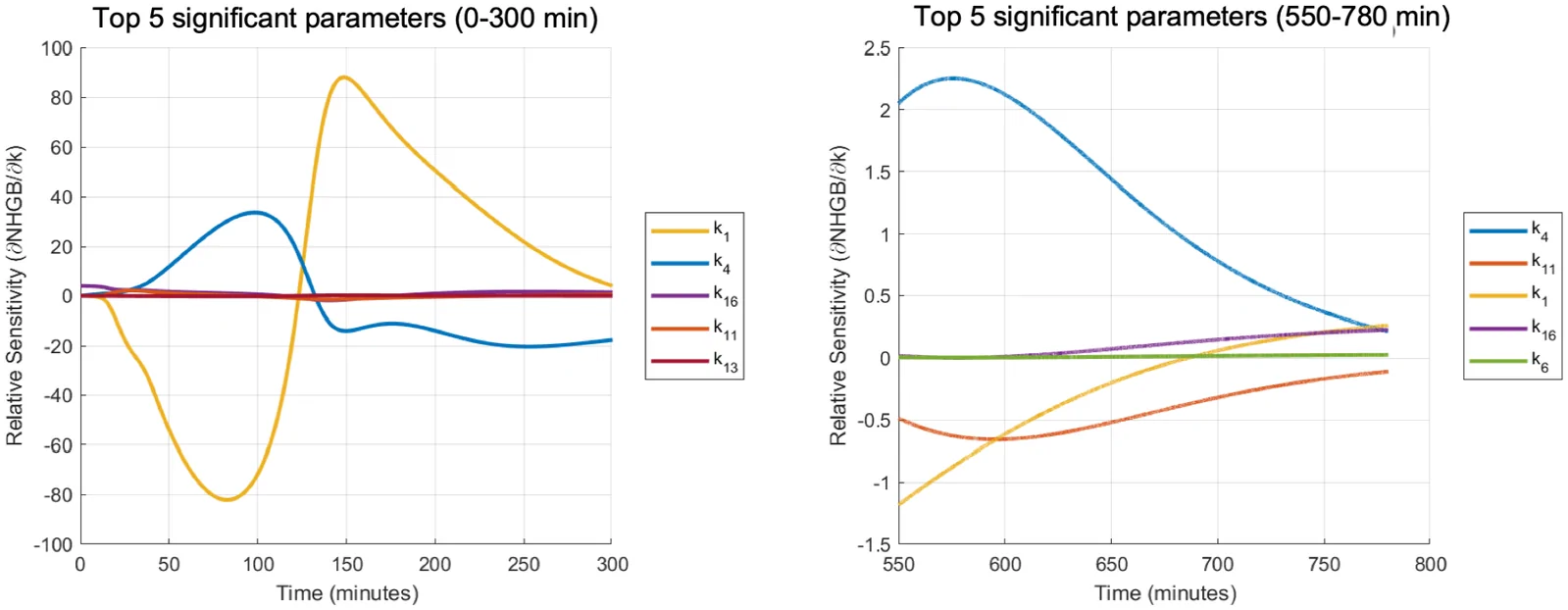
Key parameters influencing NHGB identified by sensitivity analysis. The top five parameters influencing net hepatic glucose balance during the postprandial (0–300 min) and fasting (550–780 min) periods are shown based on model sensitivity analysis. These parameters include: *k*_1_ – maximal insulin production rate; *k*_4_ – insulin clearance rate; *k*_16_ – glucagon sensitivity coefficient for hepatic glycogenolysis; *k*_11_ – maximal exercise-induced glucose uptake by muscle; *k*_13_ – insulin sensitivity coefficient for hepatic glycogenesis; and *k*_6_ – glucose concentration at which glucagon secretion reaches half-maximal output.

To further investigate the physiological factors influencing net hepatic glucose balance (NHGB), we performed a sensitivity analysis over two distinct time intervals: 0–300 minutes, representing the dynamic postprandial phase, and 550–780 minutes, reflecting fasting or near-basal regulation. Sensitivity was defined as $\frac{\partial NHGB}{\partial k}$, representing the change in NHGB induced by a unit change in parameter *k*. This analysis identifies the parameters that most significantly affect NHGB during these metabolic states. The top five parameters influencing NHGB in each time interval are shown in **Figure 5**.

As shown in **Figure 5** (left panel), during the postprandial phase (0–300 minutes), the maximum insulin production rate (*k*_1_) exhibits the largest and most dynamic influence on NHGB, with a pronounced transition from strong negative to strong positive sensitivity, indicating a time-dependent regulatory role. The insulin clearance rate (*k*_4_) also shows substantial influence, initially increasing NHGB before shifting toward a negative effect later in the postprandial period. In contrast, *k*_16_ (glucagon sensitivity for hepatic glycogenolysis), *k*_11_ (exercise-induced glucose uptake), and *k*_13_ (insulin sensitivity for hepatic glycogenesis) display low sensitivities throughout this phase.

In the fasting period (550–780 minutes; right panel), the insulin clearance rate (*k*_4_) emerges as the most influential parameter, exhibiting the highest positive relative sensitivity throughout the fasting window. It peaks at approximately 560 minutes and declines steadily afterwards. The exercise-induced glucose uptake rate by skeletal muscle (*k*_11_) exerts a modest negative influence on NHGB, which gradually diminishes over the course of the fasting period. Additionally, the maximum insulin production rate (*k*_1_) shows a dynamic influence on NHGB, with a transition from negative to slightly positive effect on NHGB over the fasting period. The glucagon sensitivity coefficient for hepatic glycogenolysis (*k*_16_) exerts a modest but progressively increasing influence on NHGB during the fasting period. While the glucose concentration at which glucagon secretion reaches half-maximal rate (*k*_6_) demonstrates minimal sensitivity across the fasting window.

### Blood glucose regulation: sensitivity analysis of influential coefficients

**Figure 6:**
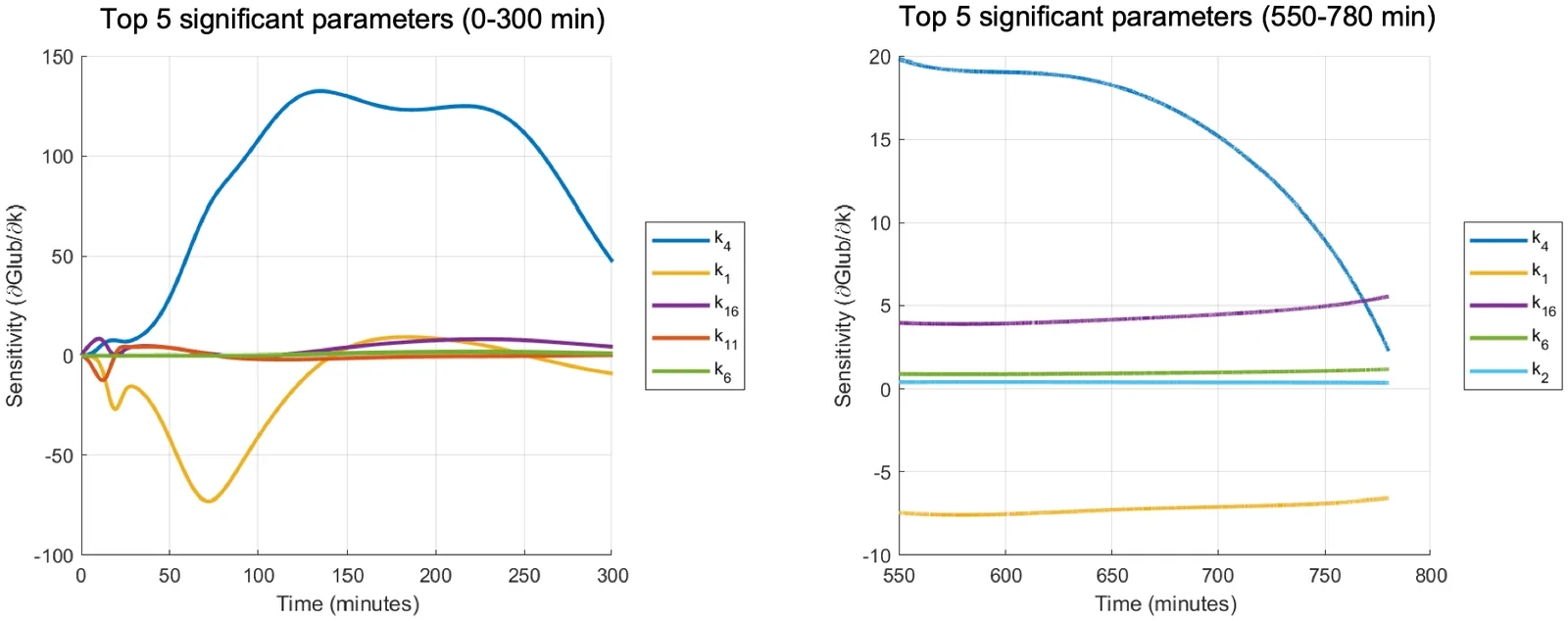
Key parameters governing blood glucose dynamics identified by sensitivity analysis. The top five parameters influencing blood glucose during the postprandial (0–300 min) and fasting (550–780 min) periods are shown based on model sensitivity analysis. These parameters include: *k*_4_ – insulin clearance rate; *k*_1_ – maximal insulin secretion rate; *k*_16_ – glucagon sensitivity coefficient for hepatic glycogenolysis; *k*_11_ – maximal exercise-induced glucose uptake by muscle; *k*_6_ – glucose concentration at which glucagon secretion reaches half-maximal output; and *k*_2_ – glucose concentration at which insulin secretion reaches half-maximal output.

As shown in **Figure 2**, both the concentration and temporal profile of blood glucose levels vary among obese, diabetic, and non-obese individuals during the postprandial period (0–300 minutes) and fasting period (550–780 minutes). To elucidate the key physiological factors governing glucose regulation during these intervals, we performed a sensitivity analysis ( $\frac{\partial Glu_{b}}{\partial k}$, *i.e*., change in *Glu_b_* induced by a unit change in parameter *k*). The top five parameters with the greatest influence on blood glucose during this fasting window are illustrated in **Figure 6**.

During the postprandial phase (0–300 minutes), glucose dynamics are dominated by insulin-mediated processes: insulin clearance rate (*k*_4_) exerts the largest positive and highly dynamic influence, while insulin secretion (*k*_1_) drives a strong opposing, glucose-lowering effect, particularly early after the meal. In contrast, glucagon sensitivity (*k*_16_) contributes a smaller but progressively increasing positive effect, whereas exercise-induced glucose uptake (*k*_11_) and the glucagon secretion threshold (*k*_6_) have comparatively minor impacts.

In the fasting phase (550–780 minutes), the insulin clearance rate (*k*_4_) exhibits the most prominent and temporally dynamic influence on blood glucose levels, while the insulin secretion rate (*k*_1_) shows a strong glucose-lowering effect. The glucagon sensitivity coefficient for hepatic glycogenolysis (*k*_16_) consistently exerts a strong glucose-raising effect. The glucose threshold for glucagon secretion (*k*_6_) has a modest positive influence on blood glucose, while the parameter *k*_2_, representing the glucose concentration for half-maximal insulin secretion, demonstrates a minimal effect on fasting glucose dynamics.

### Effects of exercise timing on glucose dynamics

**Figure 7:**
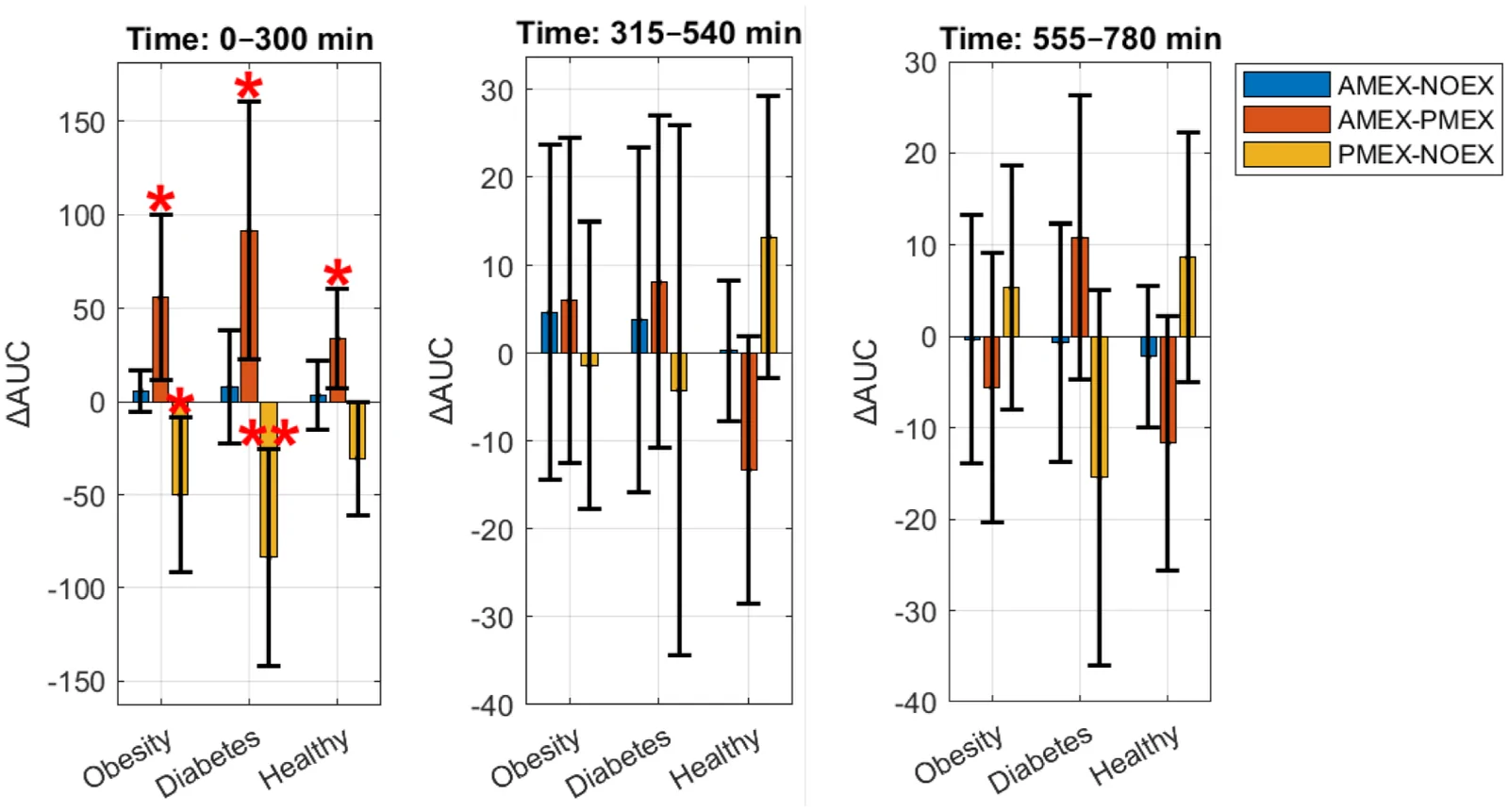
Effects of exercise timing on glucose dynamics across metabolic groups. Exercise conditions include morning exercise (AMEX), post-dinner exercise (PMEX), and no exercise (NOEX). Differences in glucose exposure are quantified as changes in area under the curve (ΔAUC) for individuals with obesity, type 2 diabetes, and non-obese groups across three time intervals: 0–300 min, 315–540 min, and 555–780 min. Pairwise comparisons are shown for AMEX vs. NOEX (blue), AMEX vs. PMEX (orange), and PMEX vs. NOEX (yellow). Positive ΔAUC values indicate higher glucose exposure in the first condition of each comparison. Error bars represent standard deviations. Red asterisks denote statistically significant differences (p< 0.05). Key finding: Post-dinner exercise (PMEX) consistently produced the lowest early postprandial glucose exposure, with the greatest benefit in individuals with obesity and type 2 diabetes.

The model was validated under three conditions: no exercise (NOEX), morning exercise (AMEX), and post-dinner exercise (PMEX). Optimized parameter estimates for each metabolic group (non-obese, obese, and diabetic) are summarized in **Tables 2**-**4**. By using the mean optimized parameter values (**Tables 2**-**4**), the model was used to simulate glucose profiles over 0–780 min post-meal. The area under the curve (AUC) was then calculated for three distinct postprandial intervals: 0–300 min, 315–540 min, and 555–780 min as done in our previous work (Kanaley et al., 2024). Paired comparisons of AUCs across exercise conditions were performed and plotted in **Figure 7**.

As shown in **Figure 7**, during the initial post-meal period (0–300 min), all subject groups exhibited significantly higher glucose elevations following morning exercise (AMEX) compared to post-dinner exercise (PMEX), as indicated by red asterisks denoting statistical significance (p< 0.05). Among individuals with obesity and diabetes, the non-exercise condition (NOEX) resulted in markedly higher glucose levels than PMEX, while non-obese subjects displayed minimal differences between NOEX and PMEX conditions. At later time intervals (315–540 min and 555–780 min), glucose responses exhibited increased variability across all groups, as indicated by the larger error bars.

To assess the impact of exercise timing on model parameters within each metabolic group, a one-way repeated-measures ANOVA was conducted separately for non-obese, obese, and diabetic individuals. This analysis tested whether the parameter values differed significantly across the three exercise conditions, while accounting for within-subject variability. The results are reported in **Tables 2**-**4**.

For the non-obese group (**Table 2**), PMEX significantly increased *k*_13_ (insulin sensitivity coefficient for hepatic glycogenesis), *k*_19_ (feedback effect of glycogen stores on hepatic glycogenolysis), and *k*_20_ (insulin sensitivity coefficient for glycogenesis in muscle), while significantly decreasing *k*_16_ (glucagon sensitivity coefficient for hepatic glycogenolysis). AMEX significantly increased *k*_19_ and *k*_20_ but did not produce the same breadth of parameter changes observed under PMEX.

For the obese group (**Table 3**), AMEX significantly increased *k*_5_ (maximum rate of glucagon secretion), *k*_13_ (insulin sensitivity coefficient for hepatic glycogenesis), *k*_14_ (glucose concentration at half-maximal hepatic glycogenesis), and *k*_19_, while significantly decreasing *k*_16_. PMEX significantly increased *k*_5_, *k*_19_, and *k*_20_, and similarly decreased *k*_16_, though it did not affect *k*_13_ or *k*_14_.

For the diabetic group (**Table 4**), AMEX significantly increased *k*_14_ (glucose concentration at half-maximal hepatic glycogenesis) and decreased *k*_16_ (glucagon sensitivity coefficient for hepatic glycogenolysis), whereas PMEX produced a significant decrease in *k*_16_ alone, suggesting a more limited parameter response to exercise in the diabetic group compared to the non-obese and obese cohorts.

## Discussion

### Analysis of model coefficient values

In comparison to non-obese individuals, the lower glucagon clearance rate in obese participants suggests a prolonged action of glucagon and elevated hepatic glucose output, a pattern previously linked to impaired counterregulatory hormone clearance in obesity (Watada and Tamura, 2017). Additionally, the reduced insulin sensitivity coefficient for hepatic glycogen synthesis and an elevated glucose concentration required for half-maximal hepatic glycogenesis indicate decreased hepatic insulin sensitivity in obese subjects (Basu et al., 2004; Staehr et al., 2004). Furthermore, the decreased insulin sensitivity coefficient for muscle glycogen synthesis in the obese group points to diminished insulin-stimulated glycogenesis in muscle, which is a hallmark of obesity-induced metabolic inflexibility (Boden et al., 2005; Czech, 2017).

Individuals with type 2 diabetes demonstrated distinct impairments in glucose regulation, particularly in parameters related to skeletal muscle glucose uptake and hepatic glucose control. The higher insulin sensitivity coefficient for muscle glucose uptake in the diabetic group contrasts with the well-established observation of insulin resistance in skeletal muscles commonly associated with type 2 diabetes (DeFronzo and Tripathy, 2009). One possible explanation for this discrepancy is that the individuals with diabetes in this study were on their glucose-lowering medications during the experimental period, which may have enhanced their apparent insulin sensitivity. A potential confounding factor in the diabetic group is the continued use of glucose-lowering medications all the diabetic participants were on. The paradoxically elevated insulin sensitivity coefficient for muscle glucose uptake (*k*_9_) in the diabetic group, which contradicts the well-established phenomenon of skeletal muscle insulin resistance in type 2 diabetes, is associated with the pharmacological enhancement of insulin action by medications. Moreover, the markedly elevated glucagon sensitivity coefficient for hepatic glycogen in the diabetic group indicates excessive hepatic glucose output - one of the key mechanisms underlying fasting hyperglycemia in diabetes.

In our model, hepatic glycogenolysis is described by using a dual-component structure (as shown in Equation 13): a classic Michaelis-Menten term that captures substrate (glycogen) saturation kinetics, and an additional feedback term governed by the parameter *k*_19_​, which introduces a compensatory acceleration in glycogen breakdown as glycogen stores decline. Together, these two components create a nonlinear relationship between glycogen concentration and glycogenolysis rate.

In non-obese individuals, the feedback parameter *k*_19_​ is nearly zero (0.0004 ± 0.0010), indicating that this compensatory mechanism plays a minimal role under normal physiological conditions. Consequently, glycogen breakdown typically slows as glycogen stores are depleted. In contrast, individuals with type 2 diabetes exhibit a significantly elevated *k*_19_ (10.8580 ± 19.8479) (p< 0.05), which introduces a strong compensatory response that can override the expected decline. Once glycogen levels fall below a certain threshold, this mechanism paradoxically accelerates glycogenolysis despite substrate limitation, contributing to persistently elevated hepatic glucose production in type 2 diabetes during fasting. This dysregulated response is consistent with observations by (Winnick et al., 2016), who reported a blunted acceleration of glycogenolysis during progressive glycogen depletion in diabetic individuals, suggesting impaired dynamic control of hepatic glucose output. The biological justification for the k_19_/Gly_l_ feedback term rests on structural and functional evidence from the glycogen biochemistry. Structurally, hepatic glycogen in type 2 diabetic animal models consists of an elevated proportion of large α-particle aggregates (interconnected β-granules) that are mechanically fragile relative to those in healthy livers (Deng et al., 2015; Besford et al., 2016). As glycogen stores are progressively depleted during fasting, these α-particles fragment into smaller β-particles, transiently increasing the density of chain termini accessible to glycogen phosphorylase, the rate-limiting enzyme in glycogenolysis, and thereby accelerating glucose release at the very moment that substrate availability (Gly_l_) is declining. Mathematically, the k_19_/Gly_l_ term produces a non-monotonic rate-versus-substrate relationship: at high Gly_l_, the k_19_/Gly_l_ contribution is negligible and glycogenolysis follows conventional Michaelis-Menten kinetics; at low Gly_l_, this term becomes dominant and accelerates breakdown beyond the rate predicted by substrate limitation alone. We emphasize that this feedback formulation represents a modeling hypothesis grounded in existing structural and functional data rather than a mechanism directly measured in the present study. The model generates a specific and experimentally testable prediction: glycogenolysis assays on liver tissue from diabetic and non-diabetic donors, conducted across a range of glycogen depletion states, should reveal divergent rate-depletion relationships consistent with the presence (diabetic) or absence (non-diabetic) of this feedback acceleration. The markedly elevated and highly variable *k*_19_ for the diabetic group (10.8580 ± 19.8479 vs. 0.0004 ± 0.0010 for non-obese controls) likely reflects genuine biological heterogeneity in glycogen structural remodeling among diabetic participants, which may depend on disease duration, glycemic control history, and medications — factors that differ substantially across individuals and are consistent with the broad phenotypic spectrum of type 2 diabetes.

The elevated muscle glucose concentration required for half-maximal glycogen synthesis in individuals with type 2 diabetes indicates a reduced sensitivity of skeletal muscle to glucose, suggesting impaired initiation of glycogen synthesis at normal physiological glucose concentrations. These findings are consistent with previous reports of defective GLUT4 translocation and disrupted insulin-mediated signaling pathways in diabetic skeletal muscle (Zierath et al., 2000).

Several key model parameters have meaningful correspondence with clinically or experimentally measurable quantities, supporting their potential translational relevance. The insulin clearance rate (*k*_4_) is functionally equivalent to the fractional hepatic insulin extraction rate, which can be estimated non-invasively from the molar ratio of C-peptide to insulin in peripheral blood, given that C-peptide and insulin are co-secreted in equimolar amounts but only insulin undergoes substantial hepatic extraction. Elevated *k*_4_, as observed in the obese group, thus indicates augmented first-pass hepatic insulin removal, which reduces the effective insulin signal reaching peripheral tissues. The glycogenolysis feedback parameter (*k*_19_) corresponds to the structural susceptibility of hepatic glycogen to progressive degradation, a property linked to α-particle fragility that has been characterized in diabetic liver tissue by electron microscopy and molecular weight analysis (Nielsen and Richter, 2003; Deng et al., 2015). These parameters should be interpreted as mechanistic descriptors inferred through system identification, providing physiologically meaningful characterization of glucose regulation and generating experimentally testable hypotheses, rather than as direct measurements of the underlying biological processes.

### Net hepatic glucose balance

Sensitivity analysis shows that insulin clearance rate exerts a positive effect on NHGB, likely because elevated insulin clearance reduces circulating insulin levels, thereby diminishing the insulin effect on glycogenesis, leading to an increase in NHGB. This mechanism is particularly relevant in conditions such as insulin resistance, where altered insulin clearance contributes to hyperglycemia (Meier et al., 2005).

The negative effect of exercise-induced glucose uptake rate by skeletal muscle on NHGB indicates that enhanced glucose uptake by muscle following physical activity can transiently suppress hepatic glucose output, particularly during the early phase of fasting. As time progresses and the metabolic effects of exercise subside, this suppression weakens.

In early fasting, higher insulin secretion suppresses hepatic glucose output, consistent with the known effects of insulin on promoting glycogenesis (Cherrington, 1999). However, during prolonged fasting, as insulin levels approach baseline, a higher maximum insulin production rate by the pancreas results in enhanced NHGB, possibly because of the nonlinear interactions between insulin and glucagon and glucagon-driven NHGB to maintain glucose availability (Basu et al., 2009).

The glucagon sensitivity coefficient for hepatic glycogenolysis has a modest but progressively increasing influence on NHGB during the fasting period, reflecting the liver’s capacity to break down glycogen into glucose. In early fasting, abundant glycogen stores support hepatic glucose output (Cherrington et al., 1979). As fasting continues and glycogen depletes, the persistent effect of *k*_16_ suggests that the liver increases the efficiency of glycogen breakdown to meet glucose demands. This rising influence may also reflect a compensatory mechanism by which the liver attempts to buffer declining glucose availability (Moore et al., 2012).

Finally, the minimal effect of glucose concentration at which glucagon secretion reaches half-maximal rate on NHGB suggests that it plays a limited role in modulating NHGB variability under steady fasting conditions. While glucagon is a critical driver of hepatic glucose production during fasting, the model indicates that variations in its glucose-response threshold have a less immediate impact compared to insulin-related parameters (Unger and Orci, 1977).

### Fasting glucose regulation: sensitivity analysis of influential coefficients

During the early phase of fasting, elevated insulin clearance markedly raises blood glucose by reducing circulating insulin availability. This attenuation of insulin action diminishes peripheral glucose uptake and suppresses glycogen synthesis in insulin-sensitive tissues, contributing to increased glycemia. As fasting progresses and insulin secretion continues to decline, the influence of insulin clearance diminishes, reflecting a shift in the dominant regulatory mechanisms of glucose homeostasis. Elevated insulin clearance has been related to impaired glycemic control and persistent hyperglycemia, particularly in individuals with insulin resistance or type 2 diabetes (Duckworth et al., 1998; Meier et al., 2005). In contrast, the insulin secretion rate shows a strong glucose-lowering effect, underscoring the fundamental role of insulin in glycemic regulation. Increased insulin secretion facilitates glucose uptake in peripheral tissues and inhibits hepatic glucose production, thereby lowering blood glucose concentrations (Cherrington, 1999; Saltiel and Kahn, 2001).

The glucagon sensitivity coefficient for hepatic glycogenolysis has a strong glucose-raising effect. Despite reduced circulating glucagon during late fasting, the liver retains high sensitivity to glucagon, sustaining glucose production via glycogen breakdown. This is consistent with previous findings demonstrating the essential role of hepatic glucagon sensitivity in maintaining euglycemia during fasting (Cherrington et al., 1979; Basu et al., 2009; Moore et al., 2012).

The glucose threshold for glucagon secretion has a modest positive influence on blood glucose. A higher threshold delays the onset of glucagon release in response to falling glucose levels, thereby limiting hepatic glucose mobilization and allowing glucose levels to rise slightly. Although glucagon secretion is diminished in prolonged fasting, the system remains responsive to threshold modulation, which may fine-tune the counterregulatory response to hypoglycemia (Gylfe and Gilon, 2014).

Finally, the minimal effect of glucose concentration for half-maximal insulin secretion on fasting glucose dynamics likely reflects the overall suppression of insulin secretion during fasting, when blood glucose levels are low and β-cell activity is markedly diminished. As a result, it plays a limited role in glucose regulation under fasting conditions (Henquin, 2000; Bratanova-Tochkova et al., 2002).

### Effects of exercise timing on glucose dynamics

The simulation results demonstrate that exercise timing exerts meaningful effects on postprandial glucose dynamics, with the direction and magnitude of these effects differing across metabolic groups. By AUC, PMEX consistently produced lower early postprandial glucose exposure than AMEX across all groups, suggesting that post-dinner exercise is more effective at attenuating glucose excursions in the immediate post-meal period. This advantage was particularly pronounced in obese and diabetic individuals, for whom NOEX also resulted in substantially higher glucose levels than PMEX, while the non-obese group showed minimal difference between NOEX and PMEX. These findings suggest that the glycemic benefit of post-dinner exercise is amplified in metabolically compromised individuals, for whom the timing of physical activity may be an especially important intervention target.

By the model parameter values, the non-obese group exhibited the broadest response to PMEX, with significant increases in *k*_13_ (insulin sensitivity coefficient for hepatic glycogenesis), *k*_19_ (feedback effect of glycogen stores on hepatic glycogenolysis), and *k*_20_ (insulin sensitivity coefficient for glycogenesis in muscle), alongside a significant decrease in *k*_16_ (glucagon sensitivity coefficient for hepatic glycogenolysis). The upregulation of *k*_13_ indicates that post-dinner exercise enhances hepatic insulin sensitivity, thereby promoting hepatic glycogen synthesis in the post-meal state. This observation aligns with previous studies demonstrating that postprandial exercise promotes hepatic glucose uptake and glycogen synthesis (De Bock et al., 2005; Frosig and Richter, 2009). The concurrent increase in *k*_19_ suggests stronger inhibitory feedback from existing glycogen stores on hepatic glycogenolysis, which may serve to limit endogenous glucose production and attenuate late postprandial glucose rises (Petersen et al., 2004; Jeppesen et al., 2007). The increase in *k*_20_ reflects enhanced skeletal muscle capacity for glycogen synthesis, in line with studies showing that exercise upregulates glycogen synthase activity and GLUT4 expression in muscle tissue, promoting more efficient glucose disposal (Booth and Winder, 2005). The reduction in *k*_16_ further indicates a diminished sensitivity of hepatic glycogenolysis to glucagon, which would reduce hepatic glucose output and contribute to the lower glucose AUCs observed under PMEX. In contrast, AMEX in the non-obese group produced a more limited parameter response, with significant increases in *k*_19_ and *k*_20_ only, suggesting that while morning exercise still promotes glycogen storage in both liver and muscle, it does not confer the same degree of improvement in hepatic insulin sensitivity or glucagon responsiveness as post-dinner exercise.

For the obese group, both AMEX and PMEX produced significant reductions in *k*_16_, indicating that either exercise time suppresses glucagon-driven hepatic glycogenolysis and thereby reduces endogenous glucose output in this group. However, the two exercise times diverged in their effects on the other parameters. AMEX uniquely increased *k*_13_ and *k*_14_ in addition to *k*_5_ and *k*_19_, suggesting that morning exercise in obese individuals promotes hepatic glycogen synthesis through both insulin-dependent mechanisms and by shifting the glucose threshold at which glycogenesis becomes active. The elevation of *k*_5_, reflecting an increased maximum rate of glucagon secretion, under both AMEX and PMEX may represent a compensatory hormonal response to exercise-induced glucose utilization (van Dijk et al., 2012; Richter and Hargreaves, 2013; Kolnes et al., 2025). Notably, PMEX increased *k*_20_, which was not observed under AMEX in this group, pointing to a timing-dependent advantage of post-dinner exercise for enhancing muscle glycogen synthesis in obesity, consistent with the lower postprandial AUCs observed under PMEX.

For the diabetic group, the parameter responses to exercise were more restricted. AMEX significantly increased *k*_14_, indicating a shift in the glucose threshold for half-maximal hepatic glycogenesis, and decreased *k*_16_, suggesting reduced glucagon sensitivity in hepatic glycogenolysis. PMEX produced only a significant decrease in *k*_16_, with no other parameters reaching the level of significance. The limited breadth of parameter changes in the diabetic group relative to the non-obese and obese cohorts likely reflects the diminished metabolic plasticity characteristic of type 2 diabetes, including impaired insulin signaling, reduced glycogen synthase activity, and attenuated hormonal responsiveness (Thivel et al., 2018; Savikj et al., 2019). Nevertheless, the consistent reduction in *k*_16_ across both exercise conditions suggests that suppression of glucagon-mediated hepatic glucose output may represent a preserved and clinically relevant pathway through which exercise exerts glycemic benefit in diabetic individuals, regardless of timing.

### Limitations and future directions

The present model is intended to be a biological characterization rather than a predictive model, as mentioned earlier. By fitting individualized parameter values to experimental data, the model enables mechanistic interpretation of inter-group metabolic differences that would be difficult to extract from conventional statistical analyses alone. However, several limitations constrain its generalizability. First, the cohort (n=52) is relatively small, and parameter estimates carry uncertainties as reflected by the CV values and standard deviations in **Tables 1**-**4**. Several maximum CV values were over 100%, indicating large uncertainties of a parameter because of one or more participants in a group. This is likely an outcome, to a large degree, of the lack of persistence in excitation. The system was excited with only a step function (meal intake) and the measured glucagon and glucose remained flat for some participants. This lack of excitation persistence limited the identifiability of some parameters for certain health conditions. Further tests with enhanced excitations are needed to validate the model. Second, the model structure was tested exclusively under controlled laboratory conditions, including a fixed meal composition and supervised exercise; thus, predictions under free-living conditions, different dietary patterns, or higher-intensity exercise would require additional validation. Third, processes not represented in the model structure, including incretin hormone dynamics, lipolysis, and *de novo* gluconeogenesis, may become relevant over longer time horizons or under different physiological perturbations. As a result of neglected dynamics and the lack of persistence in excitation, the residuals exhibited a level of bias for insulin and changes in variance, mainly for insulin and glucose (**Figure 3**). This again calls for further validation of the model with more participants and enhanced system perturbations. In addition, prospective predictive use of the model would require external validation with independent cohorts and prospective testing of model-derived recommendations. Fourth, as discussed earlier, the model coefficients revealed physiological differences while the glucose level for the diabetic participants was regulated by medications, but the potential confounding effects of medications need to be determined in further studies.

Beyond its current use as a characterization and analysis tool, the model framework has several promising translational applications. First, the identified model coefficients — particularly insulin clearance rate (*k*_4_) and the glycogenolysis feedback coefficient (*k*_19_) — could serve as soft-sensed indicators to stratify patients by metabolic phenotype. For example, individuals with markedly elevated *k*_19_ may represent a subgroup in whom glycogen structural remodeling is a dominant contributor to fasting hyperglycemia and who may benefit from therapies that target hepatic glycogen metabolism. Second, the exercise timing analysis demonstrates that the model can simulate the differential glycemic impact of morning versus post-dinner exercise across metabolic groups, suggesting its potential utility as a personalized exercise prescription tool. With further validation, the model could be adapted to generate individualized predictions of glycemic response to different exercise regimens, informing clinical exercise counseling for patients with obesity or type 2 diabetes. Furthermore, the model parameter sensitivity analysis provides a system-level framework that could complement conventional clinical assessments, offering a quantitative basis for understanding how individual differences in insulin clearance, glucagon sensitivity, and hepatic glycogen dynamics contribute to divergent glycemic phenotypes.

The present model focuses on glucose, insulin, glucagon, and glycogen metabolism, but type 2 diabetes is increasingly recognized as a systemic metabolic-network disorder involving coordinated alterations in carbohydrate, lipid, amino acid, and inflammatory metabolism, together with extensive crosstalk among the liver, skeletal muscle, adipose tissue, pancreas, and other organs. Recent metabolomics studies have demonstrated that these interconnected metabolic networks contribute to disease heterogeneity and may improve precision medicine by identifying metabolic phenotypes that are not captured by conventional clinical biomarkers (Xu et al., 2025). Future integration of metabolomic, lipidomic, and inflammatory data with the present system-identification framework may enable more comprehensive characterization of metabolic dysfunction and further improve mechanistic understanding of type 2 diabetes. Moreover, recent advances in liver metabolic phenotyping and computational hepatology have highlighted the value of integrating mechanistic models, imaging biomarkers (Ning, 2024), and artificial intelligence-assisted analyses (Zhou et al., 2026) to better characterize disease heterogeneity and improve risk stratification. Furthermore, future studies integrating body-composition measurements, nutritional and renal biomarkers, metabolomics, and other physiological data with the proposed system-identification framework may enable more comprehensive characterization of individual metabolic phenotypes and improve mechanistic interpretation of metabolic heterogeneity.

## Data Availability

The raw data supporting the conclusions of this article will be made available by the authors, without undue reservation.
